# Ordering the mob: Insights into replicon and MOB typing schemes from analysis of a curated dataset of publicly available plasmids

**DOI:** 10.1016/j.plasmid.2017.03.002

**Published:** 2017-05

**Authors:** Alex Orlek, Hang Phan, Anna E. Sheppard, Michel Doumith, Matthew Ellington, Tim Peto, Derrick Crook, A. Sarah Walker, Neil Woodford, Muna F. Anjum, Nicole Stoesser

**Affiliations:** a Nuffield Department of Medicine, University of Oxford, John Radcliffe Hospital, Oxford, UK; b NIHR Health Protection Research Unit in Healthcare Associated Infections and Antimicrobial Resistance, University of Oxford, Oxford, UK; c Antimicrobial Resistance and Healthcare Associated Infections (AMRHAI) Reference Unit, National Infection Service, Public Health England, London, UK; d Department of Bacteriology, Animal and Plant Health Agency, Addlestone, UK

**Keywords:** Replicon typing, Plasmid multilocus sequence typing, MOB typing, Antibiotic resistance, Plasmid database

## Abstract

Plasmid typing can provide insights into the epidemiology and transmission of plasmid-mediated antibiotic resistance. The principal plasmid typing schemes are replicon typing and MOB typing, which utilize variation in replication loci and relaxase proteins respectively. Previous studies investigating the proportion of plasmids assigned a type by these schemes (‘typeability’) have yielded conflicting results; moreover, thousands of plasmid sequences have been added to NCBI in recent years, without consistent annotation to indicate which sequences represent complete plasmids. Here, a curated dataset of complete Enterobacteriaceae plasmids from NCBI was compiled, and used to assess the typeability and concordance of *in silico* replicon and MOB typing schemes. Concordance was assessed at hierarchical replicon type resolutions, from replicon family-level to plasmid multilocus sequence type (pMLST)-level, where available. We found that 85% and 65% of the curated plasmids could be replicon and MOB typed, respectively. Overall, plasmid size and the number of resistance genes were significant independent predictors of replicon and MOB typing success. We found some degree of non-concordance between replicon families and MOB types, which was only partly resolved when partitioning plasmids into finer-resolution groups (replicon and pMLST types). In some cases, non-concordance was attributed to ambiguous boundaries between MOBP and MOBQ types; in other cases, backbone mosaicism was considered a more plausible explanation. β-lactamase resistance genes tended not to show fidelity to a particular plasmid type, though some previously reported associations were supported. Overall, replicon and MOB typing schemes are likely to continue playing an important role in plasmid analysis, but their performance is constrained by the diverse and dynamic nature of plasmid genomes.

## Highlights

• 92% of clinically-relevant plasmids could be replicon typed, compared with reports of 100% typeability in 2014.
• Replicon and MOB typing were partly concordant; partitioning plasmids into finer-resolution groups increased concordance.
• Overlap between MOBP and MOBQ relaxase families complicates assignment of MOBP and MOBQ types.
• Generally, resistance genes showed low fidelity towards particular plasmid backbones.
• PacBio sequencing has driven increased availability of complete plasmid sequences, but retrieved datasets require curation.

## 1 Introduction

Plasmid genomes generally consist of a somewhat conserved ‘backbone’ of genes associated with functions such as replication and transfer, accompanied by variable sets of ‘accessory genes’. Backbone loci have been exploited as phylogenetic markers to group plasmids (Garcillán-Barcia and de la Cruz, 2013). Accessory genes often confer adaptive traits, notably, antibiotic resistance (Partridge, 2011). Plasmid-mediated resistance dissemination is common amongst the Enterobacteriaceae family of gram-negative bacteria, which includes clinically important taxa such as *Escherichia coli* and *Klebsiella* spp. (Iredell et al., 2016). Of particular concern is the rise in resistance to β-lactam antibiotics, frequently driven by plasmid-borne genes including extended-spectrum β-lactamase (ESBL) genes (e.g. *bla*_CTX-M_), as well as carbapenemase genes (e.g. *bla*_KPC_) (Livermore and Woodford, 2006, Nordmann et al., 2012). Transmission of resistance gene-carrying plasmids (‘resistance plasmids’) can drive the success of recipient strains (Holt et al., 2013), so it is important to understand resistance plasmid epidemiology, as well as strain epidemiology.

Plasmid typing can provide insights into resistance plasmid epidemiology (de Been et al., 2014, Leverstein-van Hall et al., 2011, Orlek et al., 2017, Pecora et al., 2015), such as whether resistance dissemination involves diverse plasmids or one dominant ‘epidemic’ type (Valverde et al., 2009). Some resistance genes have been associated predominantly with specific replicon types (Akiba et al., 2016, Carattoli, 2009, Carattoli, 2013), for example, *bla*_OXA-48_ with IncL/M (Poirel et al., 2012). However, the extent to which reported associations reflect recent local expansion of a resistance plasmid, or stable widespread associations remains unclear. If the latter, then plasmids stably harbouring resistance genes could potentially be targeted using sequence-specific antimicrobials (Bikard et al., 2014, Williams and Hergenrother, 2008). Other applications of plasmid typing include plasmid detection; notably, distinguishing plasmid from chromosomal contigs in short-read *de novo* assemblies (Lanza et al., 2014).

The most widely used plasmid classification schemes are replicon and MOB typing, which exploit loci encoding plasmid replication (replicons) and mobility functions (relaxases), respectively (Alvarado et al., 2012, Carattoli et al., 2005, Carattoli et al., 2014, Garcillán-Barcia et al., 2009). Replicons include various different loci, none of which are universal across plasmids (del Solar et al., 1998), whereas relaxases are thought to be universally present amongst plasmids that mobilise via the relaxase-*in*-*cis* mechanism (Garcillán-Barcia et al., 2011, Ramsay et al., 2016). However, relaxase homology can be distant, even amongst plasmids of the same MOB type (Garcillán-Barcia et al., 2009). Replicon types can be assigned by querying plasmids against various replicon sequences using BLASTN (Carattoli et al., 2014), whilst MOB types can be assigned using profile-based searches such as PSI-BLAST; *in silico* probes representing each relaxase family are used to query a dataset of plasmids, and thereby assign MOB types (Francia et al., 2004, Garcillán-Barcia et al., 2009, Guglielmini et al., 2011). Six probes have been used to detect relaxases of Gammaproteobacterial plasmids, whilst a further two probes have been used to detect relaxases in other taxa (Guglielmini et al., 2011). PSI-BLAST can uncover distant homology, so MOB typing provides a lower resolution but potentially more inclusive classification (Altschul et al., 1997, Garcillán-Barcia and de la Cruz, 2013).

Within the replicon typing framework, plasmids can be classified at hierarchical resolutions: for common replicon types, plasmid multi-locus sequence typing (pMLST) schemes have been devised for sub-typing (Brolund and Sandegren, 2015, Hancock et al., 2016), whilst replicon types also belong to broader replicon families. Most replicon families were originally defined according to plasmid incompatibility, which is a manifestation of the genetic relatedness of replicons (Taylor et al., 2004). *In silico* analysis of replication initiation protein genes has indicated that based on sequence similarity, the traditional incompatibility families represent phylogenetically-coherent groups (Carattoli et al., 2014). However, the Col ‘family’ of plasmids was defined according to the more superficial phenotype of colicin production, and it has long been known that this so-called ‘family’ includes phylogenetically diverse plasmids (Riley and Gordon, 1992).

A key limitation of replicon typing is that individual plasmids can contain multiple replicons, complicating classification, whereas usually just one relaxase is encoded (Garcillán-Barcia and de la Cruz, 2013). However, due to its higher resolution, replicon typing provides more detailed information on plasmid relatedness, particularly if a pMLST scheme is available (Pallecchi et al., 2007). MOB typing only types relaxase-encoding plasmids, which constitute varying proportions of total plasmids across different taxa (Guglielmini et al., 2011, Smillie et al., 2010); replicon typing is most developed for Enterobacteriaceae-associated plasmids. The proportion of plasmids that can be assigned to a type using the schemes (‘typeability’) is likely to be influenced by biases in the sequencing datasets on which typing is conducted, as well as the bioinformatic approaches used. In 2014, *in silico* replicon typing was reported to type all publicly available clinically-relevant Enterobacteriaceae plasmids (Carattoli et al., 2014). Smillie et al. (2010) reported that around half of Gammaproteobacterial plasmids could be assigned a MOB type. In contrast, Shintani et al. (2015) found only 75% of publicly available Enterobacteriaceae plasmids could be replicon typed, and 44% of Gammaproteobacterial plasmids could be MOB typed (see Table S1 in Shintani et al., 2015); however, bioinformatic methods differed from those originally proposed.

Making accurate epidemiological inferences using plasmid typing depends on the ability of a typing scheme to assign phylogenetically-coherent types to plasmids (Belkum et al., 2007). For replicon and MOB typing schemes to reflect phylogenetic relationships accurately, they should be concordant - that is, replicon families should nest within the broader-resolution MOB type families. Previous studies have supported concordance, with several exceptions (see Fig. 5 in Garcillán-Barcia et al., 2011). Non-concordance could result from backbone mosaicism (Hiraga et al., 1994, Kulinska et al., 2008), frequently due to recombination (Bianco and Kowalczykowski, 1997, Osborn et al., 2000). Alternatively, non-concordance may reflect fuzzy boundaries between the MOB families. Indeed, there is known to be some level of overlap between the MOBP and MOBQ families such that PSI-BLAST searches with MOBP and MOBQ probes sometimes uncover the same homologs (Garcillán-Barcia et al., 2009). However, non-concordance has not been the focus of previous studies so it remains unclear which explanation is most likely.

Re-investigating the performance of replicon and MOB typing is timely given the increasing numbers of publicly available complete plasmid sequences (Conlan et al., 2014). In this study, we curated a dataset of all complete publicly available Enterobacteriaceae plasmids to allow an up-to-date, comprehensive assessment of replicon and MOB typing schemes in terms of typeability and concordance. Concordance was assessed at hierarchical resolutions from replicon family-level to pMLST-level where available. Associations between plasmid replicon types and resistance genes were also examined.

## 2 Methods

### 2.1 Retrieving complete plasmid sequences and associated metadata from NCBI

Putative complete plasmid accessions were retrieved from the NCBI nucleotide database (https://www.ncbi.nlm.nih.gov/nucleotide/) on 26th August 2016, using an Entrez query with filters to exclude some incomplete or non-plasmid accessions at this stage (Supplementary methods S1). Duplicate sequences (those sharing 100% sequence identity with another retrieved sequence) were removed, preferentially retaining RefSeq over GenBank accessions (Pruitt et al., 2007), and more richly annotated over less richly annotated Genbank accessions. Biopython scripts (Cock et al., 2009) were used to retrieve information and filter accessions, including filtering-out non-coding sequences, and eliminating incomplete plasmid sequences using a regular expression search of accession title descriptions (Supplementary methods S1). Multi-locus sequence typing (MLST) using all available Enterobacteriaceae schemes (http://pubmlst.org/data/) was also conducted to identify and remove chromosomal sequences mis-annotated as plasmids, using BLAST as described (Larsen et al., 2012). Additional manual filtering involved examining putative plasmids at the tails of the sequence length distribution, to remove accessions thought to represent chromosomal sequences or partial plasmid sequences (Supplementary methods S1).

EDirect (Kans, 2013) was used to retrieve accession metadata, including the ‘completeness’ annotation which was used to guide filtering. Edirect was also used to retrieve additional metadata not used for filtering, including the sequencing technology, and the ‘create date’ of the accession in NCBI (Nahin, 2008). *In silico* replicon typing using the PlasmidFinder database has been designed and assessed according to a selection of clinically-relevant plasmids (Carattoli et al., 2014). Therefore, to make direct comparisons with analyses of original authors, plasmids in the quality-filtered dataset were assigned as clinical or non-clinical according to the source taxa. Specifically, genus and, where necessary, species-level information on host organism and human pathogenicity derived from the PATRIC database was used to guide assignments (Wattam et al., 2014).

### 2.2 Replicon typing and pMLST, MOB typing, resistance gene detection

For replicon typing, the complete plasmid sequences were queried against a locally downloaded version (retrieved 20th April 2016) of the PlasmidFinder database (http://www.genomicepidemiology.org/), using recommended percentage identity and coverage thresholds of 80% and 60% respectively (Carattoli et al., 2014). Where multiple hits aligned to the same locus (defined by an overlap spanning 50% of the length of the shorter sequence), best hits were selected according to percentage identity and coverage. *In silico* pMLST was conducted for IncF, IncN, IncA/C, IncHI1, IncHI2 and IncI1 plasmids (García-Fernández et al., 2008, García-Fernández et al., 2011, García-Fernández and Carattoli, 2010, Hancock et al., 2016, Phan et al., 2009, Villa et al., 2010). pMLST was conducted using locally downloaded allele databases (http://pubmlst.org/plasmid/) with recommended identity and coverage thresholds of 85% and 66% (Carattoli et al., 2014). For each allelic locus, the best hit was again selected according to percentage identity and coverage.

To perform MOB typing, PSI-BLAST searches (Altschul et al., 1997) were conducted using N-terminal relaxase protein sequences as queries against a database of complete plasmid sequences, translated in all six frames. Searches were run for ≤ 14 iterations - the maximum number of iterations used by previous authors (Garcillán-Barcia et al., 2009). Initially, we used E-value thresholds in accordance with those used previously. However, we found that certain plasmids previously assigned a MOB type, were left unassigned (Supplementary methods S1, Supplementary methods S2). As the calculation of E-values accounts for database size, the E-value associated with a given hit is inflated when BLAST searching against a larger database (Jones and Swindells, 2002). We hypothesised that this could account for the discrepancy in MOB typing. Hence, to optimise the E-value threshold, we used a set of plasmids for which MOB typing had previously been conducted and validated (Supplementary methods S1, Supplementary methods S2). Based on this, we chose E-value thresholds as follows: MOBC, 0.001; MOBF, 0.01; MOBH, 0.01; MOBP, 1; MOBQ, 0.0001; MOBV, 0.01. After hits were produced by PSI-BLAST, no identity or coverage thresholds were applied (allowing for distant homologies to be detected); however, stringent filtering was used to select best hits (Supplementary methods S1). To investigate the robustness of our MOB typing results, we re-ran PSI-BLAST searches using a different set of six MOB query proteins representing each MOB family (Supplementary methods S1, Supplementary methods S2).

Resistance genes were detected by BLAST querying the ResFinder database, using recommended identity and coverage thresholds of 98% and 60% respectively (Zankari et al., 2012). Best hits were selected as described above for replicon typing.

### 2.3 Visualisation and statistical analysis

All analyses were performed using R (https://www.r-project.org/). The circlize package (Gu et al., 2014) was used to create chord diagrams to visualise associations between replicon families/types/sub-types and MOB types using an edgelist as input. A given replicon type and MOB type detected on the same plasmid were represented as a single edge. An equivalent approach was used to visualise associations between replicon families/replicon subtypes/MOB types and a selection of key β-lactamase genes prevalent in our dataset.

In chord diagram visualisations of plasmid typing data, if multiple replicons of the same family/type were detected on a given plasmid, the plasmid type was considered to be the set of unique families/types detected. That is, a plasmid typed as IncFIB, IncFIC, IncFIC would be represented as IncFIB, IncFIC at the replicon type level, and as IncF at the family level. Likewise, for MOB typing, a MOBF, MOBF plasmid would be represented simply as MOBF in visualisations.

Predictors of plasmid typing success were investigated visually and using statistical analysis. Binary logistic regression was used to investigate whether plasmid size (log10-transformed) and the number of detected resistance genes (all resistance genes included; values truncated at the 95th percentile to reduce outlier influence) independently predicted plasmid typeability by each scheme. Associations between replicon families/MOB types and prevalent β-lactamase genes were investigated using Fisher's exact test, with p-values computed by Monte Carlo simulation.

## 3 Results and discussion

### 3.1 Characteristics of the curated plasmid dataset

A total of 6952 sequences representing putative Enterobacteriaceae plasmids were retrieved from the NCBI nucleotide database. Deduplicating identical sequences (2815 removed) and programmatically filtering probable non-plasmid/partial plasmid sequences (1892 removed) left 2245 accessions. Of these, 2063 met inclusion criteria (Supplementary methods S1), whilst the remaining 182 accessions were further examined to decide upon inclusion or exclusion; 148 were excluded according to Methods described above (104 not annotated as ‘complete’, 32 found to contain MLST loci, 12 manually filtered) leaving 2097 complete Enterobacteriaceae plasmids for analysis (Supplementary Table S1). The plasmid sequence files have been made publicly available, and could be utilized in various areas of plasmid research (Orlek et al., in press).

**Fig. 1:**
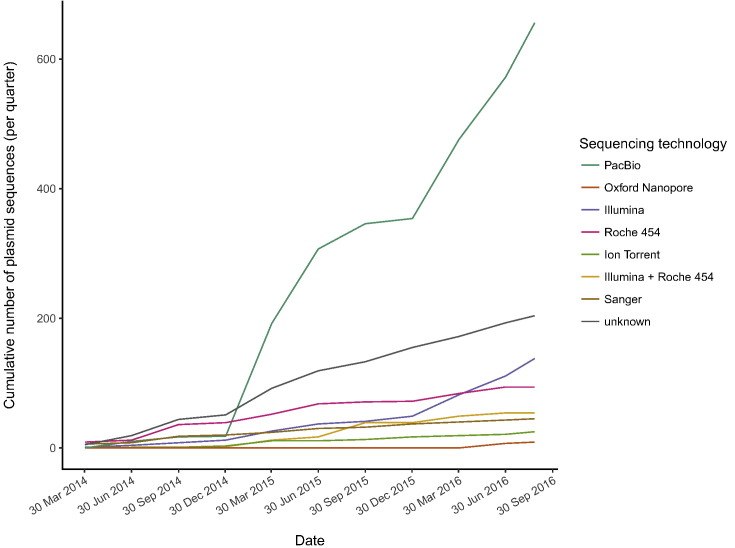
Complete plasmid accessions added to NCBI since 2014. Where a hybrid short-read/long-read sequencing approach was used (as indicated by the accession metadata), this is represented as long-read only.

Plasmids ranged in size from 1.3 kb to 794 kb. The plasmid size distribution was log-bimodal (Supplementary Fig. S1) as reported previously (Shintani et al., 2015). The source organisms were dominated by human pathogens, especially *E*. *coli*, *K*. *pneumoniae*, and *S*. *enterica* (Supplementary Fig. S2). PacBio sequencing technology has clearly been a key driver of the increase in complete plasmid sequences since 2014 (Fig. 1; data prior to 2014 not shown due to a lack of annotation for sequencing technology). The first complete plasmids sequenced using Oxford Nanopore technology were added in June 2016 (Both et al., 2016) and this technology will likely drive further expansion in availability of complete plasmid sequences in future.

### 3.2 Assessment of database curation methods

Sequence topology annotation (circular/linear) was an unreliable indicator of complete plasmid sequences, with 57 of the 2097 curated plasmids annotated as ‘linear’. At least one of these accessions represents a genuine linear plasmid (accession NC_011422) (Baker et al., 2007) but remaining accessions may represent mis-annotations, since ‘linear’ is set as the default value on submission (Fetchko and Kitts, 2011). Not counting accessions excluded as duplicates, 190 excluded accessions were annotated as ‘circular’. Of these, 27 were excluded due to MLST allele detection (indicating an accession was likely chromosomal), and four were excluded following manual inspection. The majority of the other excluded ‘circular’ accessions were filtered due to being described as ‘whole genome shotgun’ sequences which should only be used to describe incomplete genomes (NCBI, 2014). Additional manual review of these accessions might have identified some that would warrant inclusion as complete plasmids.

### 3.3 Assessment of typing performance: typeability

**Fig. 2:**
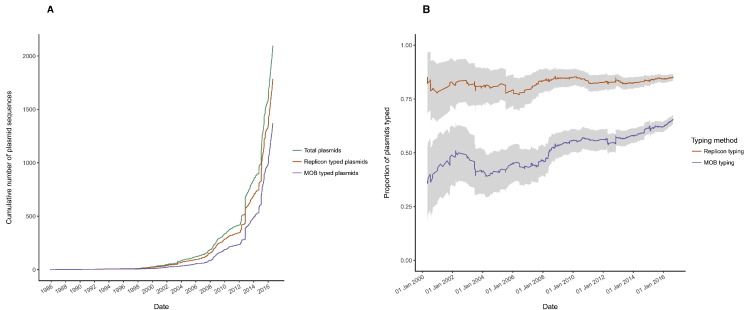
(A) Cumulative number of plasmids added to NCBI from initial accession (7th November 1985) to sequence retrieval date (26th August 2016). Cumulative counts reflect all plasmids (green), as well as the subsets that were replicon typed (red) and MOB typed (blue). (B) Proportion of plasmids added to NCBI prior to a given date that could be typed by replicon typing (red) and MOB typing (blue). Grey shading around the lines represents a 95% binomial confidence interval, calculated by the Agresti-Coull method.

Over 1000 complete Enterobacteriaceae plasmids have been added to NCBI in the past two years (Fig. 2A), underscoring the need to re-assess the performance of plasmid typing schemes. Overall, a replicon type was detected in 1784 (85%) plasmids whilst a MOB type could be assigned to 1371 (65%) plasmids; together the schemes typed 1872 (89%) plasmids (Supplementary Table S1). When typeability was assessed over time (based on the date on which an accession became available), the proportion of plasmids replicon typed remained relatively constant, whilst the proportion of plasmids MOB typed tended to increase (Fig. 2B). This increase may reflect a bias in the size of available plasmid accessions over time (Supplementary Fig. S3); specifically, larger plasmids which became available later were also more likely to be MOB typed (see Section 3.4). Previous authors reported in 2010 that around half of publicly available plasmids from Gammaproteobacteria - the taxonomic class to which the Enterobacteriaceae family belongs - could be MOB typed (Smillie et al., 2010). In comparison, we were able to assign MOB types to a greater proportion of plasmids. However, the increase in proportion of MOB-typeable plasmids over time would seem to explain this discrepancy (Fig. 2B); of the plasmids in our dataset that were available in 2010, roughly half could be assigned a MOB type, in agreement with previous authors. Our finding that only 85% of plasmids could be replicon typed contrasts with results of Carattoli et al., who demonstrated 100% typing success. We wanted to determine the extent to which this discrepancy may reflect differences in the taxonomic scope of our analysis, relative to that of Carattoli et al., who used a selection of clinically-relevant Enterobacteriaceae plasmids to design and assess *in silico* replicon typing. When plasmids from non-clinical taxa were excluded from our analyses (in line with Carattoli et al.), 1675/1818 (92%) plasmids were assigned replicon types (Table S1). The remaining disparity largely reflects incomplete classification of plasmid sequences added to NCBI in the two years since *in silico* replicon typing was devised (Supplementary Fig. S4A). Thirty-eight clinical plasmids lacking a replicon type were submitted prior to 2014, but were not included in the dataset used by Carattoli et al., perhaps due to temporary suppression of these records in NCBI (Pruitt et al., 2010).

One advantage of MOB typing lies in its ability to detect divergent plasmid backbones not detected by the replicon typing scheme (Garcillán-Barcia and de la Cruz, 2013). Supporting this, 88 plasmids were MOB typed, but not replicon typed. Interestingly, of these 88 plasmids, non-clinical plasmids were over-represented (47% versus 13% of plasmids in the whole dataset), which may reflect the fact that *in silico* replicon typing has only been validated for clinical plasmids (Carattoli et al., 2014).

Relative to Shintani et al. (75% and 44% Enterobacteriaceae plasmids replicon and MOB typed respectively), we found greater typeability for both schemes. To investigate the discrepancy, our analysis pipeline with E-value thresholds optimised for this dataset (Supplementary methods S1) was applied to the 926 Enterobacteriaceae plasmids used in the assessment by Shintani et al. (see Table S1 in Shintani et al., 2015). When typing was conducted (as described in Methods), 81% and 48% of plasmids were replicon and MOB typed respectively. When filtering steps were applied (as described in Methods), 66 sequences were excluded; 83% and 48% of the remaining plasmids were replicon and MOB typed respectively (Table S1). Therefore, the discrepancy between typing performance reported here and in the study by Shintani et al. largely reflects the different bioinformatic methods used to implement the typing schemes (e.g. Shintani et al. used TBLASTN rather than PSI-BLAST to detect relaxases), and highlights the importance of using consistent methodological approaches when undertaking comparisons across datasets.

Another aspect of typeability is the extent to which the types detected are unambiguous; more specifically, when multiple types are detected on the same plasmid, this makes it difficult to interpret the phylogenetic affiliation of the plasmid, especially when these types belong to different families of replicon/MOB type. Multi-replicon types are thought more common than multi-type MOB types, and this was also supported in our dataset. Of the 1784 plasmids replicon typed in total, 502 (28%) contained multiple replicons, of which 167 (9% of the total) represented plasmids with replicons belonging to different replicon families. In comparison, of the 1371 plasmids MOB typed in total, only 58 (4%) contained multiple relaxases, and of these, 31 (2% of the total) were plasmids with relaxases belonging to different MOB types.

Typeability of the pMLST schemes was also assessed (Supplementary Fig. S5). pMLST was conducted on plasmids belonging to an unambiguous replicon type, for which a pMLST scheme was available; overall, pMLST was conducted on 868/1784 (48%) replicon typed plasmids. Of these 868 plasmids, 82% could be assigned a known pMLST type. With the exception of the IncHI1 scheme (represented by only six plasmids), the pMLST schemes did not achieve 100% typeability. Failure to assign a pMLST type was either due to no alleles being detected, or the detected allele(s) not corresponding to a known allelic profile. Our findings suggest that plasmid backbones are more diverse than envisaged when the pMLST schemes were originally devised.

### 3.4 Association between plasmid characteristics and typeability

**Fig. 3:**
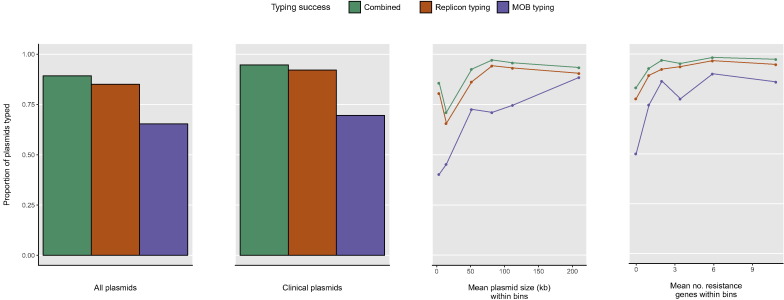
Relationship between typing success and different plasmid characteristics. For plasmid size, bins are equally sized (kb) 1.3–6.1; 6.1–35; 35–68; 68–95; 95–137; 137–794. For number of resistance genes (all resistance classes included), bin ranges and sizes are: 0, *n* = 1089; 1, *n* = 311; 2, *n* = 134; 3–4, *n* = 192; 5–8, *n* = 183; 9–34, *n* = 194.

Typeability below 100% does not necessarily invalidate a classification scheme. One way to assess the usefulness of the typing schemes is by assessing their typeability in relation to plasmid characteristics that may be of most interest. Such characteristics include size (large plasmids being more likely to be conjugative, or encode accessory genes conferring important phenotypes); presence of antibiotic resistance genes; and coming from taxa with clinical relevance. As only 13% plasmids came from non-clinical taxa, clinical status was not investigated as a predictive factor in multivariate analyses. Size and number of resistance genes were univariably (Fig. 3) and multivariably associated with typeability; logistic regression analysis showed that plasmid size and number of resistance genes were significant independent predictors of plasmid typing success (Table 1). The odds of a plasmid being replicon typed were 1.49 times higher per log_10_ kb increase in plasmid size and 1.28 times higher per additional resistance gene. The odds of a plasmid being MOB typed were 2.82 times higher per log_10_ kb increase in plasmid size and 1.2 times higher per additional resistance gene (Table 1). Therefore, plasmids for which a replicon or MOB type could be assigned were generally larger and/or encoded more resistance genes.

**Table 1:** Logistic regression analysis of plasmid typing success.

Explanatory variables	B (SE)^a^	Odds ratio (95% CI)^b^
Model of replicon typing success		
Plasmid size (log_10_ kb)	0.40 (0.10)⁎⁎⁎	1.49 (1.22–1.81)
Number of resistance genes	0.25 (0.04)⁎⁎⁎	1.28 (1.19–1.39)
Model of MOB typing success		
Plasmid size (log_10_ kb)	1.04 (0.09)⁎⁎⁎	2.82 (2.38–3.34)
Number of resistance genes	0.18 (0.02)⁎⁎⁎	1.20 (1.14–1.26)

a B coefficients are weights associated with explanatory variables; SE is standard error.

b Odds ratios indicate the change in odds resulting from a unit change in the value of the explanatory variable. Values > 1 indicate that as the value of the explanatory variable increases, the odds of typing success increase.

⁎⁎⁎ Indicates p < 0.0001.

**Fig. 4:**
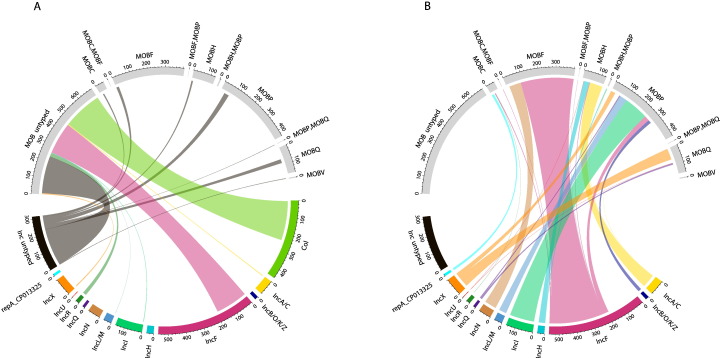
Chord diagrams illustrate associations between replicon families and MOB types; circularly arranged sectors represent replicon families and MOB types, and scale bars indicate their relative sizes. Associations are indicated by intersecting chords. Replicon family sectors, coloured; MOB type sectors, grey. (A) Replicon family-MOB type associations amongst plasmids un-typed by one or both schemes. (B) Replicon family-MOB type associations amongst plasmids with both replicon and MOB type detected. Data on Col plasmids are deliberately not shown (see main text). Plasmid types are represented as the unique set of families detected on a plasmid, as described in Methods (i.e. IncF, IncF = IncF). Where multiple types of different replicon families are detected, data are not shown, but are presented in Fig. S6. Replicon family types detected in fewer than 10 plasmids are not shown except where a non-concordant pattern of association is observed (IncU) or where associated with a multi-type MOB type.

Fig. 4A shows that the typeability of MOB typing varied considerably between plasmids belonging to different replicon families. The majority of Col plasmids were not assigned a MOB type. This may be linked with the small average size of Col plasmids (mean size ~ 6.7 kb) combined with the particularly poor typeability of MOB typing relative to replicon typing for plasmids of this size (Fig. 3).

### 3.5 Assessment of typing performance: concordance

The schemes showed a degree of phylogenetic concordance, with some replicon families nested entirely within a single MOB type, consistent with MOB typing being a phylogenetically broader scheme. Col plasmids are not shown at the family level, since non-concordance is to be expected; Col plasmids include phylogenetically distinct plasmids, and do not encode a single replicon type. Indeed, Col plasmids replicate by different mechanisms: in some cases, a plasmid-encoded Rep protein initiates replication; in other cases (ColE1 and ColE1-like plasmids) replication is initiated by binding of a transcript called RNAII, and inhibited by antisense RNAI (del Solar et al., 1998). Accordingly, the *in silico* replicon typing scheme targets several different Col plasmid replicon loci, including RNAI-encoding loci as well as loci encoding RepA replication proteins Carattoli et al., 2014; these proteins in turn belong to distinct protein families (Supplementary Table S4). Of the remaining plasmids that were investigated at the family level, IncA/C, IncF, IncH, IncQ, IncU and IncX replicon families did not show complete concordance (Fig. 4B); in cases where only a few replicon families nest outside a primary MOB type (e.g. IncA/C) non-concordance is more clearly observed by inspecting Supplementary Table S1. Replicon family–MOB type associations involving plasmids with multiple different replicon families detected are shown separately (Fig. S6); patterns of association correspond with those of the constituent single family replicons. For example, IncA/C, IncN type was associated with MOBF, MOBH type, consistent with Fig. 4B (IncN associated with MOBF and IncA/C primarily associated with MOBH). Compared with previous reports of non-concordance for just IncQ and IncP families and associated MOB types (Garcillán-Barcia et al., 2011), we find more widespread non-concordance, although we do not find non-concordance for IncP, presumably reflecting the narrower taxonomic scope of this study, in which IncP plasmids were infrequent.

**Fig. 5:**
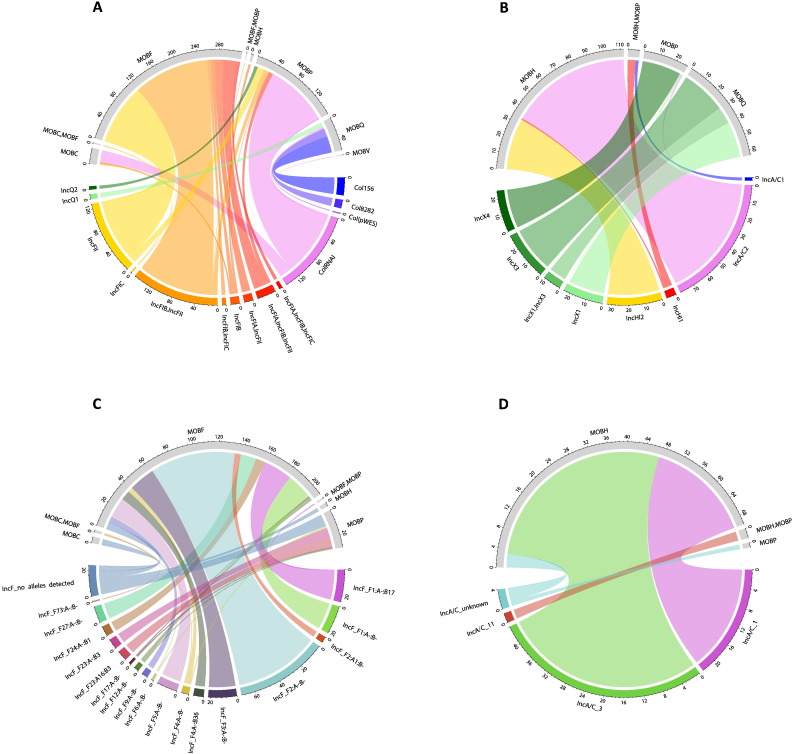
Chord plots A and B illustrate associations between replicon and MOB types, for replicon types belonging to non-concordant replicon families. Where replicon types are shown to be non-concordant and a pMLST scheme is available, chord plots C and D show associations between pMLST types and MOB types; IncHI1 pMLST associations are not shown due to the small number of plasmids belonging to this subtype. Plasmid types are represented as the unique set of types detected on a plasmid, as described in Methods (i.e. IncFIA, IncFII, IncFII = IncFIA, IncFII). Replicon types and pMLST types detected in fewer than 5 plasmids are not shown except where a non-concordant pattern of association is observed, or where associated with a multi-type MOB type. Associations with untyped plasmids are not shown. Col family replicon types correspond to the replicon probe names in the PlasmidFinder database. IncF pMLST is based on the so-called FAB formula where sequence types are determined according to the allele for IncFII, IncFIA and IncFIB. As an example, the most common IncF pMLST type is F2:A-:B- which reflects detection of allele 2 for IncFII, and detection of no alleles for IncFIA and IncFIB.

There are several explanations for the patterns of non-concordance in Fig. 4B, as described previously. To investigate whether non-concordance reflects fuzzy boundaries between MOB families, we examined PSI-BLAST hits to identify cases where different MOB query proteins aligned to the same locus on a given plasmid. This would indicate that the relaxase detected at that locus showed homology to different MOB queries, reflecting overlap between the corresponding MOB protein families; this could in turn result in non-concordance between typing schemes. There were 204 loci from 201 plasmids involved in such alignments and the MOB queries involved were all MOBP/MOBQ (Supplementary Table S2). This finding reiterates previous reports of overlap between MOBP and MOBQ families and is supported by subsequent analysis in this study with a different set of MOB queries (see Section 3.6). Accordingly, MOBP/Q overlap could potentially explain the pattern of non-concordance observed for IncQ and IncX replicon families, which each associate with both MOBP and MOBQ. It could also explain the non-concordance of ColRNAI and Col(pWES) replicon types (Fig. 5A).

To gain additional insight, we further examined alignments involving different MOB queries at the same locus. If the best two hits at a locus involve different MOB queries, the assigned MOB type is presumably less reliable, compared with a situation where top hits involve the same query; therefore, MOBP/Q overlap would seem a more plausible explanation for non-concordance in the former compared with the latter situation. Applying this reasoning to our data, we found that IncX plasmids were commonly associated with (putatively) less reliable MOB type assignments, as was one ColRNAI plasmid (accession NC_013509.1) (Supplementary Table S2). This ColRNAI plasmid, having MOBQ and subsequently MOBP as top hits, corresponds to the single MOBQ-associated ColRNAI plasmid represented in Fig. 5A. In contrast, for IncQ plasmids, although there are cases where different queries align to the same locus, the top five hits at a locus consistently involve the same MOB query (Supplementary Table S2); this suggests MOB type assignments for IncQ plasmids are reliable. These findings are supported by analyses in Section 3.6.

Given our findings, for IncQ plasmids, as well as remaining replicon families not associated with MOBP and MOBQ, backbone mosaicism provides a more plausible alternative explanation for non-concordance (Osborn et al., 2000). To investigate further, we examined the extent to which non-concordance could be resolved by partitioning plasmids into finer resolution replicon/pMLST types. If non-concordance stems from mosaicism that arose deep in plasmid evolutionary history, partitioning plasmids into more homogenous groups might mean such groups would show concordance.

Fig. 5A and B show that partitioning plasmids into finer-resolution replicon types resolves non-concordance for the IncQ family, with IncQ1 and IncQ2 associated with MOBQ and MOBP respectively. This reflects a previous study (Garcillán-Barcia et al., 2011), and is in line with the known biology of IncQ plasmids. Specifically, IncQ2 plasmids are known to encode a mobilization system unrelated to IncQ1 plasmids but resembling that of IncP plasmids, indicating backbone mosaicism (Rawlings and Tietze, 2001). For other replicon families, some replicon types show concordance, but ColRNAI and Col(pWES) remain non-concordant as do IncA/C2, IncHI1, and many of the IncF replicon types. IncX replicon types are largely concordant, but this is unlikely to be attributed to resolution of mosaicism (see Section 3.6).

Concordance is further improved by partitioning replicon types into pMLST types (Fig. 5C and D). However, some IncF pMLST types show non-concordance: F17:A-:B-, F12:A-:B-, F6:A-:B- and F4:A-:B-, F1:A-:B17. Non-concordant typing of IncF plasmids is perhaps unsurprising given the available literature on plasmid backbone mosaicism. Specifically, a study of IncFII replicons demonstrated mosaicism, with homology to the closely-related IncB, IncK and IncZ replicons (treated as IncB/O/K/Z in the Carattoli et al. *in silico* scheme), putatively due to recombination events (Praszkier et al., 1991). In our dataset, IncB/O/K/Z is associated with MOBP (Fig. 4B) as are some IncFII replicon types (Fig. 5A), providing an explanation for IncFII non-concordance. Overall, the finding of non-concordance across hierarchical resolutions for IncF replicons indicates that non-concordance in this family may in part reflect backbone mosaicism generated by relatively recent evolutionary events.

### 3.6 Assessment of the robustness of MOB typing results, using alternative MOB queries

PSI-BLAST searches against the same database but using different queries may retrieve a different set of hits (Bhagwat and Aravind, 2007). Therefore, to assess the robustness of our MOB typing results, we conducted MOB typing using an alternative set of six MOB queries representing each MOB type. 54% of plasmids were MOB typed - this is lower than achieved in our original analysis, and only 6 plasmids not previously assigned a MOB type could be MOB typed using the alternative prototypes. Additional results are presented in Supplementary Figs. S8–S11. Overall, the re-analysis supports principal findings from our primary analyses which suggests that our conclusions are robust: for example, typing schemes were again found to be partially concordant, and concordance improved when plasmids were partitioned into higher resolution replicon types/sub-types.

However, there are some key differences in the assignment of plasmid types using original vs. alternative sets of MOB queries, as summarised in Supplementary Table S3. Where a plasmid is assigned a different MOB type using the original *vs*. alternative set of queries, this indicates that the MOB type assignment may be unreliable. Crucially, such discrepancies provide a complementary way to assess the conclusions in Section 3.5 regarding MOBP/Q overlap as an explanation for non-concordance of the IncX replicon family and Col accession NC_013509.1. Overall, plasmids assigned a MOBQ type with original queries were more likely to be assigned a MOBP type using alternative queries (there are 94 plasmids for which this is the case). This underscores the ambiguous boundaries between MOBP and MOBQ families. Of plasmids assigned a different MOB type using alternative queries, the majority belong to IncX family. When typing with alternative queries, the IncX replicon family is entirely nested within MOBP, whereas primary analysis demonstrated non-concordance at the family-level, with the majority of IncX replicon types, except for IncX4, associated with MOBQ. One Col plasmid (NC_013509.1) was assigned a different MOB type using alternative queries; this is the same Col plasmid identified in Section 3.5 as having an unreliable MOB type. Also in support of conclusions in Section 3.5, there were no discrepancies in the typing of IncQ plasmids. Overall, this supports conclusions that MOBP/Q overlap can complicate typing, and cause non-concordance between replicon and MOB typing schemes, as we conclude is the case for IncX non-concordance in Fig. 4B.

### 3.7 Associations between plasmid types and resistance genes

**Fig. 6:**
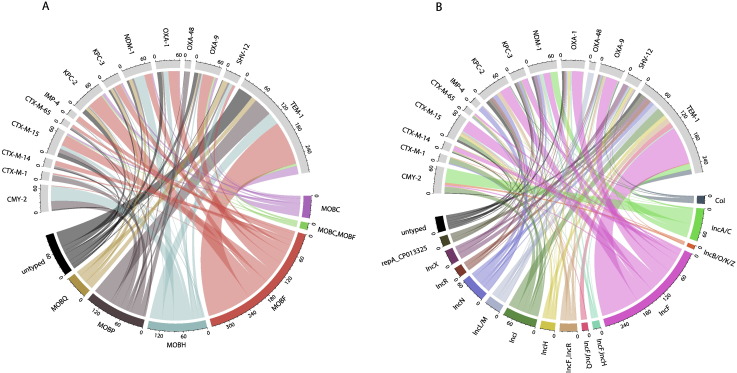
Associations between β-lactamase resistance genes identified and (A) MOB types; (B) replicon families. MOB types/replicon families detected in fewer than 10 plasmids are not shown.

IncF plasmids appeared to play a key role in shuttling major β-lactamase resistance genes, including *bla*_CTX-M-14_ and *bla*_CTX-M-15_ (Fig. 6). IncA/C was strongly associated with *bla*_CMY-2_ and to a lesser extent *bla*_NDM-1_. *bla*_OXA-48_ was associated with MOBP/IncL/M, as previously reported (Poirel et al., 2012). Overall though, plasmid backbones were associated with a variety of resistance genes, as expected, given widespread multi-drug resistance. This generally held true even when looking at pMLST types (Supplementary Fig. S6). When associations at the replicon family/MOB type level were investigated statistically, the null hypothesis that resistance genes were distributed randomly amongst plasmid types was rejected (p < 0.0001). However, this result should be interpreted cautiously given the likelihood of biases in the plasmid accessions submitted to NCBI.

## 4 Conclusions

Retrieval and curation of a dataset of complete plasmids and associated metadata from NCBI requires bioinformatics expertise. Without such a dataset, even the most basic aspects of plasmid biology, such as the size distribution, are obscured. Overall, our experience suggests that obtaining a high-quality dataset of complete plasmids requires some degree of quality-filtering, and this is likely to become more important as more sequences are added to NCBI. However, our curation methods were guided by the annotations (and mis-annotations) observed in the retrieved dataset. Building on and fine-tuning the suggested curation methods, as publicly available databases expand, would provide a valuable resource for future research. Indeed, curated plasmid datasets have a wide range of applications beyond investigation of typing schemes (Orlek et al., in press).

This study demonstrated that 11% of Enterobacteriaceae plasmids available in mid-2016, could not be typed by either replicon or MOB typing schemes. As many more plasmids are sequenced, including from a wider diversity of strains (especially strains harbouring small plasmids or plasmids with few resistance genes) typing success may decrease. Overall, replicon typing covers a wider diversity of Enterobacteriaceae plasmids than MOB typing. However, it is unclear whether novel replicon probes can be found to detect currently non-typeable plasmids. Furthermore, interpreting typing success from a dataset of complete plasmid genomes does not reflect the context in which *in silico* typing tools are often used. Plasmids can be difficult to assemble accurately from reads generated by widely used short-read sequencing technology, resulting in contig assemblies of variable quality, which may impede successful typing (Clausen et al., 2016, Inouye et al., 2014).

Although previous studies have examined plasmid backbone mosaicism and the concordance of typing schemes, as well as associations between resistance genes and plasmid types, this has not been based on comprehensive bioinformatic analysis at hierarchical typing resolutions. Our findings demonstrate that plasmid typing is inherently difficult and even the relatively conserved loci used for major typing schemes exhibit phylogenetic discordance, seen most notably with the IncF replicon. In this case, partitioning plasmids into more homogenous groups using pMLST improved concordance, but only partially, perhaps reflecting mosaicism resulting from relatively recent evolutionary events.

Our study has also highlighted issues with reproducibility of typing results, in particular, MOB typing results. To conduct replicon typing, plasmids are searched against the PlasmidFinder database, which has remained relatively static in size and content since first developed, and minor changes are recorded (https://cge.cbs.dtu.dk/services/PlasmidFinder/); hence replicon typing results should be reproducible. On the other hand, MOB typing, has traditionally been conducted by querying known MOB proteins against a database of plasmids (and sometimes also chromosomes, if integrative conjugative elements are of interest), using profile-based methods such as PSI-BLAST. Differences in database size and content can influence MOB typing results. In practice, especially when MOB typing a handful of plasmids, standard non-iterative BLAST has been commonly used (Dziewit and Bartosik, 2014, Shintani et al., 2015); however, this approach is likely to be less powerful, since position-specific information about conservation of relaxase protein residues amongst a dataset of plasmids is not harnessed. Our curated plasmid dataset is publicly available and could be used as a basis for conducting more reproducible MOB typing (Orlek et al., in press), whilst harnessing information contained within the large dataset. The ConjDB database, which includes relaxase proteins (Guglielmini et al., 2013), could also be used for this purpose. Our analysis also demonstrates that the overlap between MOBP and MOBQ families can complicate MOB typing; MOBP and MOBQ types should therefore be treated with particular caution, and ideally, the robustness of MOBP/MOBQ type assignments should be validated using different MOB queries.

We found that resistance genes tended not to show strong fidelity towards particular plasmid backbones, although the association between IncL/M and *bla*_OXA-48_ was supported. The patterns of association observed should be interpreted cautiously since they reflect biases in the NCBI database. An alternative approach might be to assess associations between plasmid backbones and resistance genes within specific geographical or temporal contexts of interest, for example by using BioSample metadata (Barrett et al., 2012, Federhen et al., 2014). If associations between specific resistance genes and plasmid backbones hold across broad timeframes or geographies this would represent stronger evidence for genuinely stable associations. More generally, combining genomic epidemiology (plasmid typing and resistance gene detection) with BioSample metadata could also provide contextual epidemiological information for a given plasmid (i.e. whether it is an isolated case or part of an outbreak). As more plasmid sequence data become available, this kind of analysis could become increasingly useful (Chang et al., 2016, Nolte et al., 2015).

In summary, ‘Ordering the mob’ denotes not only the challenging pursuit of plasmid typing, but also the process of obtaining a dataset of complete plasmids from NCBI. Using our curated plasmid dataset, we demonstrate that current typing schemes fail to classify the complete diversity of plasmids, and that there is a degree of non-concordance between replicon and MOB typing schemes. In some cases, non-concordance is likely to reflect the plasticity (and consequent mosaicism) of plasmid genomes, whilst in other cases it reflects the ambiguous boundaries between MOBP and MOBQ types.

The following are the supplementary data related to this article.

Supplementary figures Figs. S1–S3 describe the curated plasmid dataset; Fig. S4 is related to Fig. 2 (main text) but is based only on plasmids from clinically-relevant taxa; Fig. S5 shows typeability of the pMLST schemes; Fig. S6 illustrates associations between multi-type replicon families and MOB types; Fig. S7 shows pMLST/resistance gene associations; Figs. S8–S11 show results produced when MOB typing is conducted using the alternative set of MOB queries. Image 1

Supplementary methods S1 Methods for compiling the curated plasmid dataset are described. In addition, details on downloaded datasets used for plasmid typing and MLST are provided. MOB typing methods are outlined, including the sequences of query proteins, and bioinformatic procedures for selecting best PSI-BLAST hits. Supplementary methods S1

Supplementary methods S2 Methods used to select optimal PSI-BLAST E-value thresholds for MOB typing are demonstrated. Supplementary methods S2

Table S1 Details of the curated plasmids are provided including assigned replicon and MOB types, and detected resistance genes. The plasmids that were filtered at different stages of the curation process are shown. Results of applying our analysis pipeline to Enterobacteriaceae plasmids previously analysed by Shintani et al. are also shown. Table S1

Table S2 PSI-BLAST alignments prior to best hit selection are shown. Hits involving different MOB queries aligning to the same locus on a given plasmid are indicated. Table S2

Table S3 MOB typing results produced with alternative MOB queries are compared with results produced from original MOB queries. Table S3

Table S4 The zip file contains a spreadsheet file showing the conserved domains associated with replication proteins of the PlasmidFinder database. The relationship between replicon types in terms of sharing of replication protein domains is visualised using networks. The networks are provided as separate files within the zip file. The interactive network (html file) opens in a web browser and is a bipartite network - one set of nodes represents replicon types, another set of nodes represents conserved domains. The other network is a static network (PDF file) derived from the bipartite network; it shows replicon type nodes only. Table S4
