# Supplementary material for: Ordering the mob: Insights into replicon and MOB typing schemes from analysis of a curated dataset of publicly available plasmids
Source: Plasmid. 2017 May;91:42–52. doi: 10.1016/j.plasmid.2017.03.002 (PMC5466382; doi:10.1016/j.plasmid.2017.03.002)
Supplement: Supplementary figures — Figs. S1–S3 describe the curated plasmid dataset; Fig. S4 is related to Fig. 2 (main text) but is based only on plasmids from clinically-relevant taxa; Fig. S5 shows typeability of the pMLST schemes; Fig. S6 illustrates associations between multi-type replicon families and MOB types; Fig. S7 shows pMLST/resistance gene associations; Figs. S8–S11 show results produced when MOB typing is conducted using the alternative set of MOB queries. [file mmc1.docx]

## **Supplementary Figures**

### Supplementary Figure S1


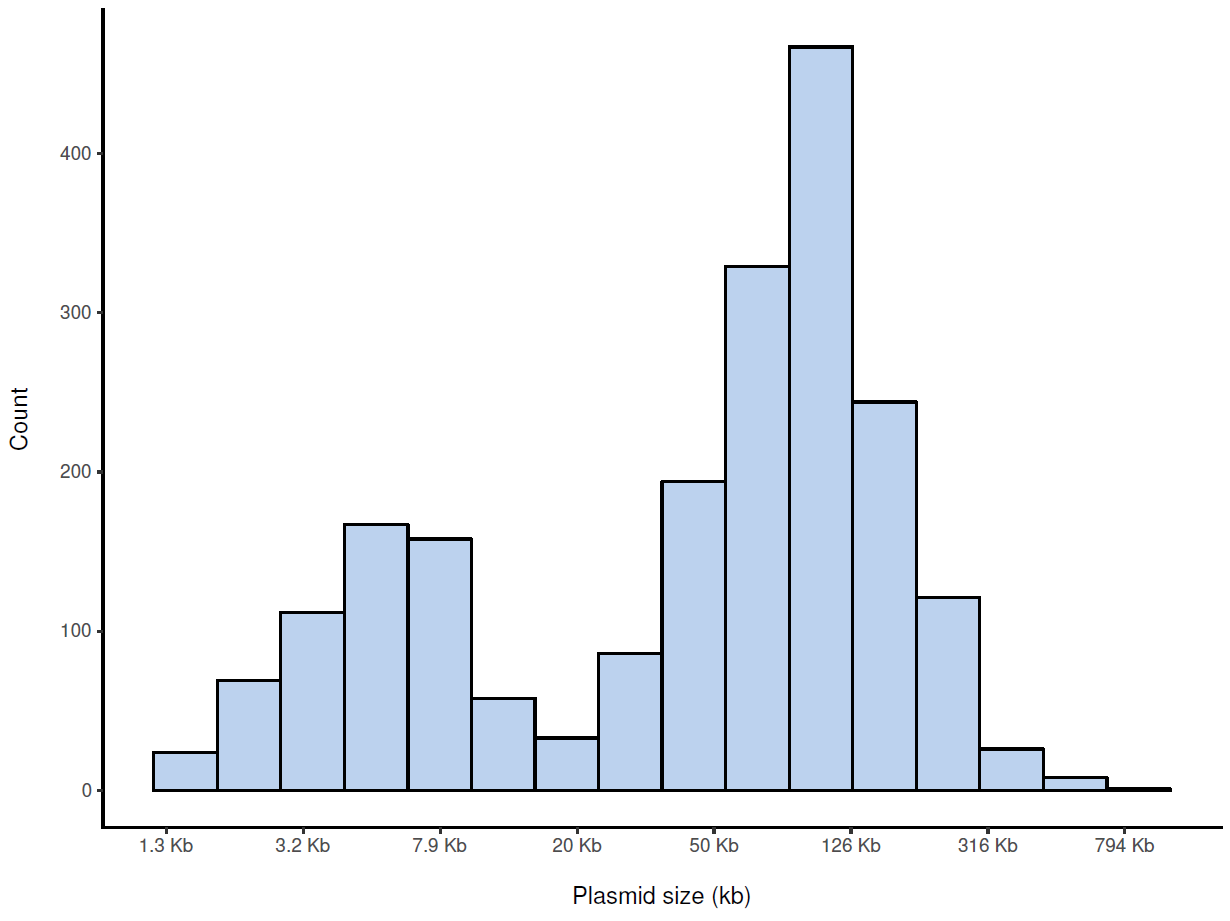


Figure S1 Histogram showing plasmid size distribution based on the 2097 curated plasmids. Plasmid size is on a log_10_ logarithmic scale, and axis values are shown in terms of kilobase pairs.

### Supplementary Figure S2


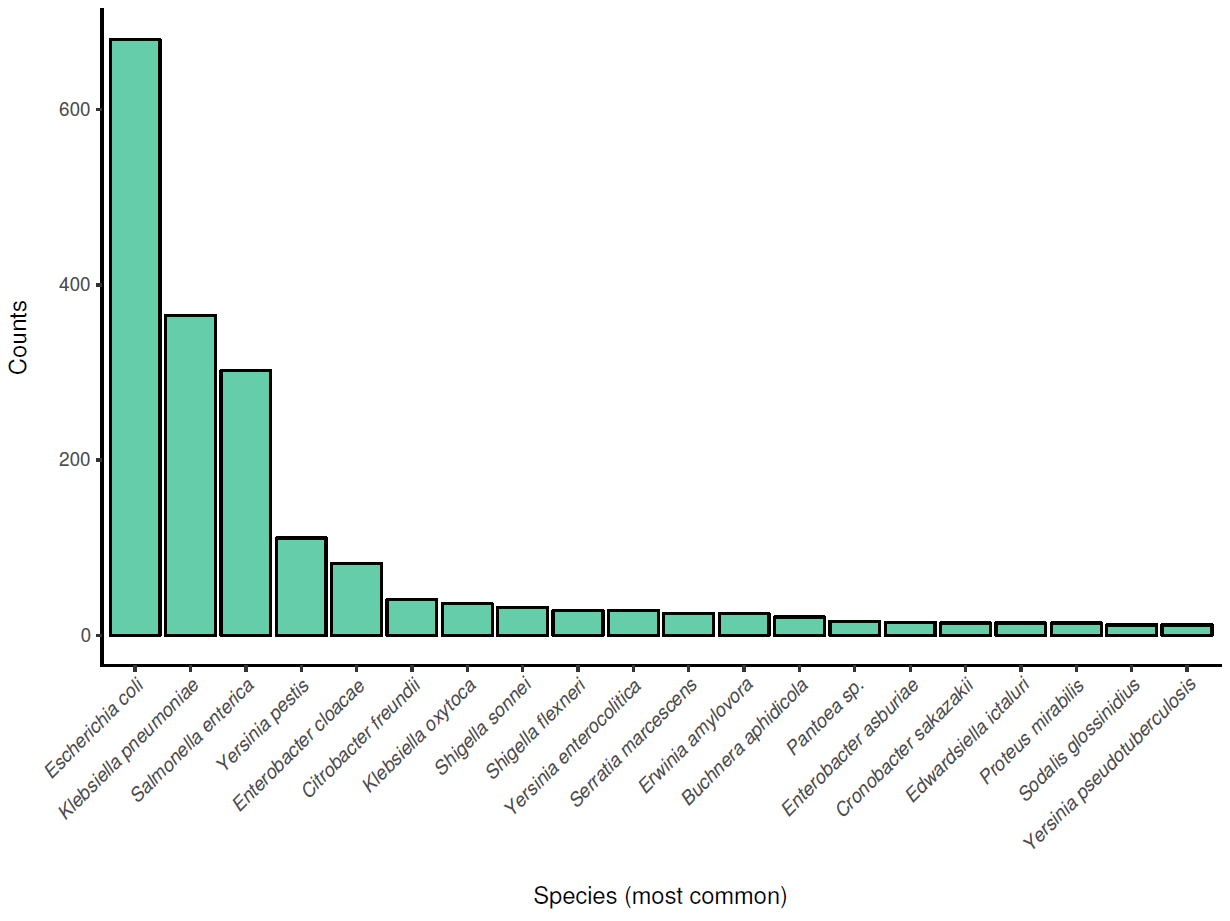


Figure S2 Frequency plot showing the most frequent source species from which the 2097 curated plasmids are derived. Only the top 20 most frequent species are shown.

### Supplementary Figure S3


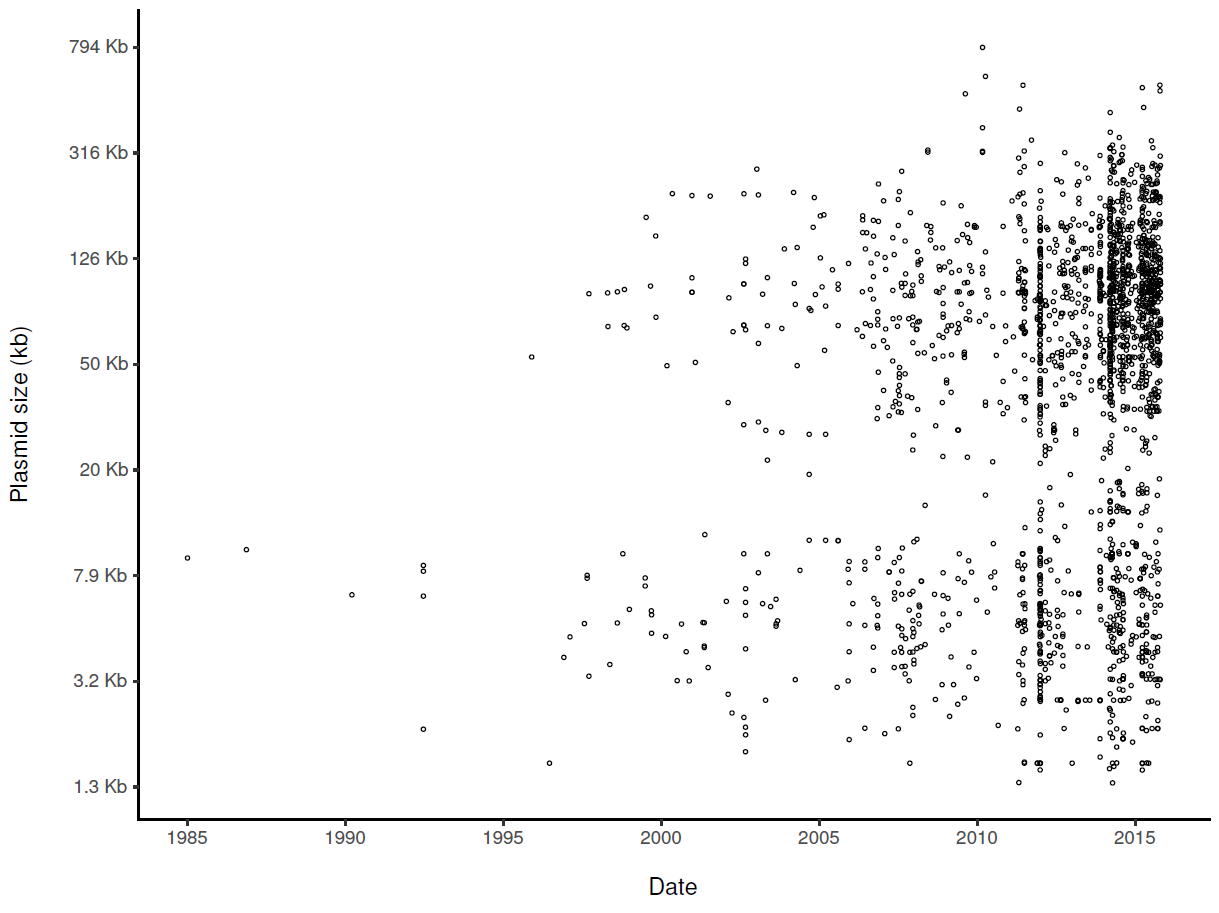


Figure S3 Plasmid size in relation to the date on which a plasmid was added to NCBI. Plasmid size is on a log_10_ logarithmic scale, and axis values are shown in terms of kilobase pairs.

### Supplementary Figure S4


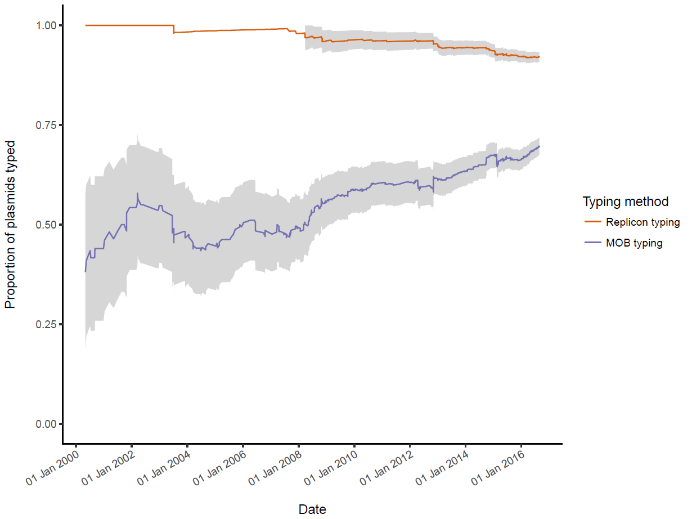

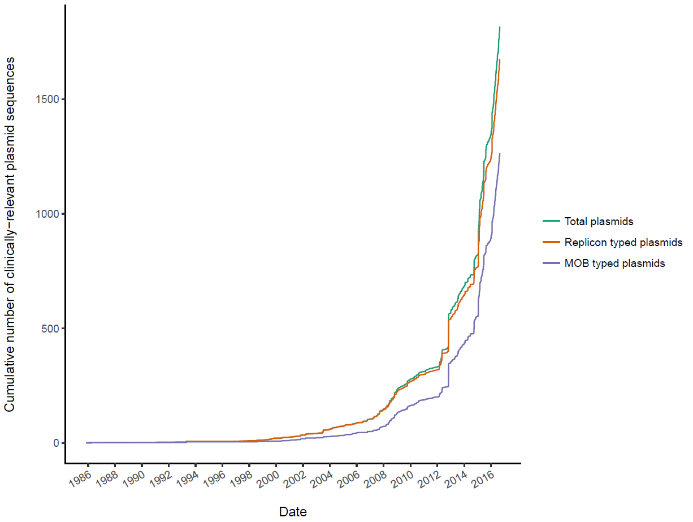


**A**

**B**

Figure S4 (A) Cumulative number of clinical plasmids added to NCBI from date of initial accession (7th November 1985) to sequence retrieval date (26th August 2016). Cumulative counts reflect all clinical plasmids (green), as well as the subsets which are replicon typed (red) and MOB typed (blue). (B) Proportion of clinical plasmids, added to NCBI prior to a given date, that can be typed by replicon typing (red) and MOB typing (blue). Grey shading around the lines represents a 95% binomial confidence interval, calculated by the Agresti-Coull method. Clinical plasmids as opposed to non-clinical plasmids were defined according to methods presented in the main text. Categorising clinical status allows comparison with the analysis conducted by Carattoli et al. in 2014 (for details see main text).

### Supplementary Figure S5


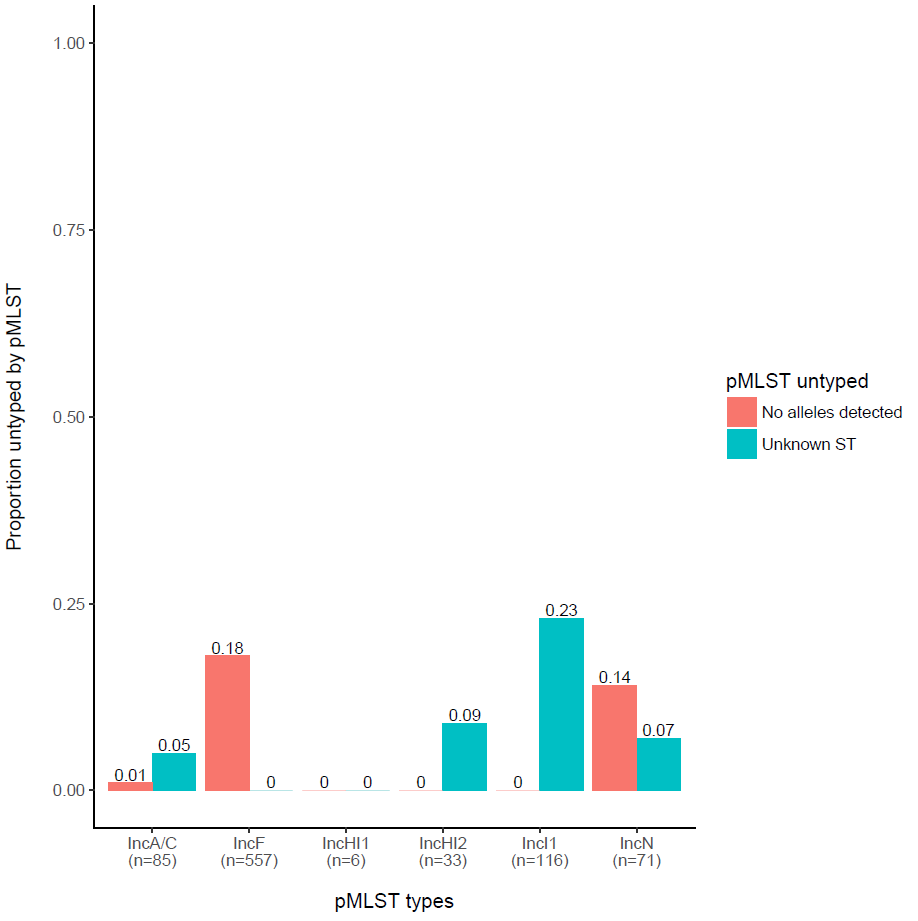


Figure S5 Proportion of plasmids that could not be assigned to a pMLST type following pMLST typing using a given scheme. Failure of pMLST typing may be due to no alleles being detected (pink) or detected alleles not matching a known allelic profile (turquoise). The number of plasmids on which pMLST typing using a given scheme was conducted is indicated on the x-axis. Values above bars indicate proportions. Note that IncF pMLST cannot produce an unknown ST since STs are assigned according to the FAB formula rather than being based on correspondence to a recognised allelic profile.

### Supplementary Figure S6


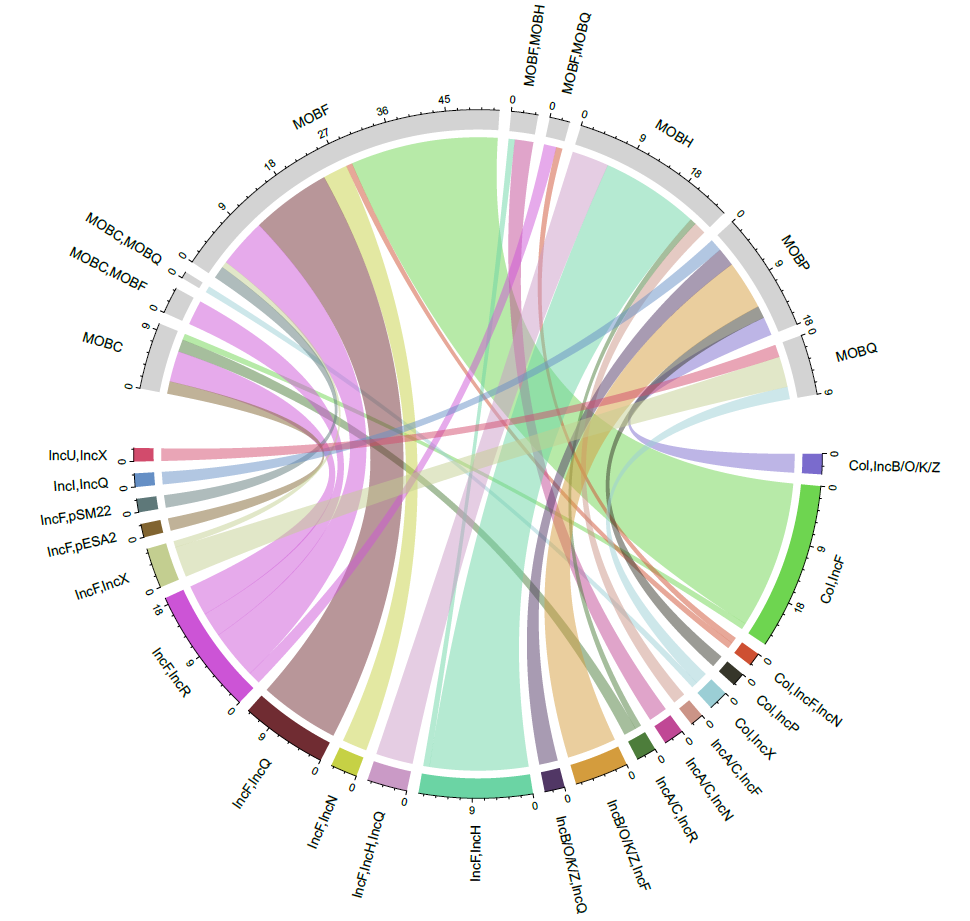


Figure S6 Chord diagram illustrating associations between multi-type replicon families and MOB types; circularly arranged sectors represent multi-type replicon families and MOB types, and scale bars indicate their relative sizes. Associations are indicated by intersecting chords. Replicon family sectors, coloured; MOB type sectors, grey. For details see main text.

### Supplementary Figure S7

Figure S7 Associations between prevalent β-lactamase resistance genes and replicon subtypes as determined by pMLST. (A) IncA/C plasmids (B) IncF plasmids (C) IncHI2 plasmids (D) IncI1 plasmids (E) IncN plasmids. Note that IncHI1 plasmids are not shown due to the small numbers of plasmids belonging to this subtype. pMLST types detected on fewer than 5 plasmids are not shown.


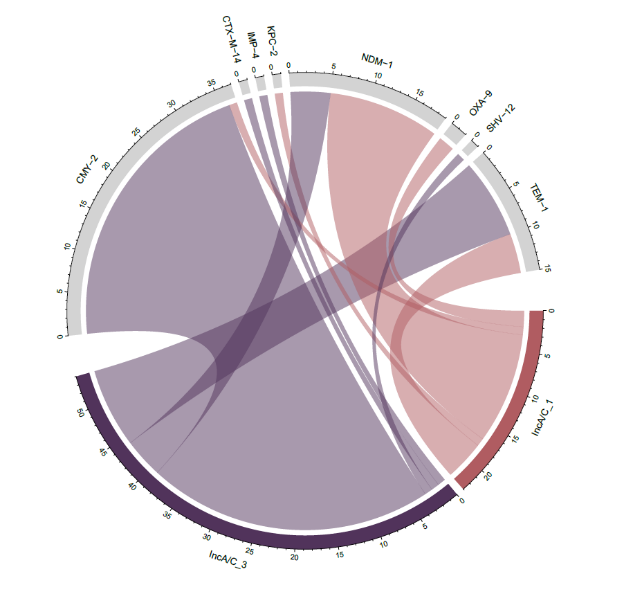

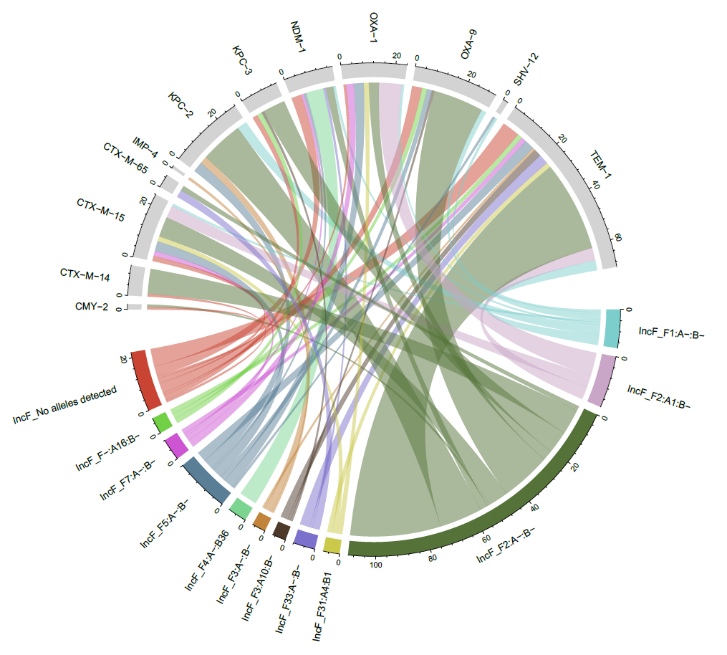


**A**

**B**


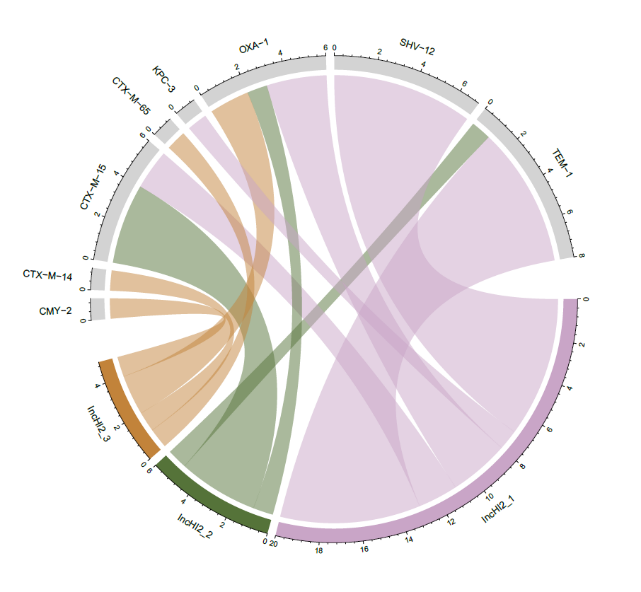

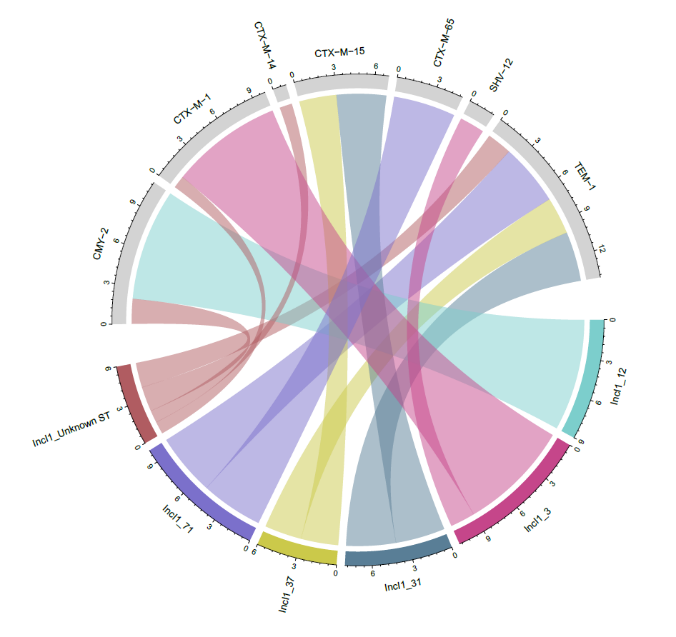


**C**

**D**


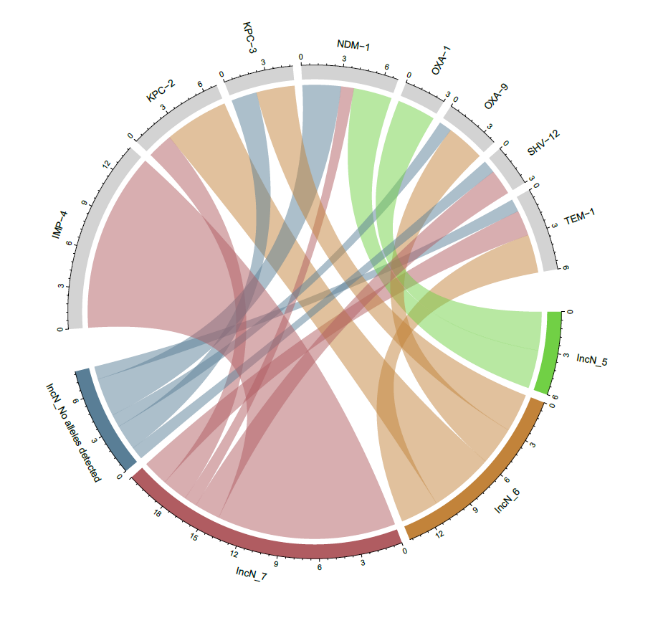


**E**

### Supplementary Figure S8


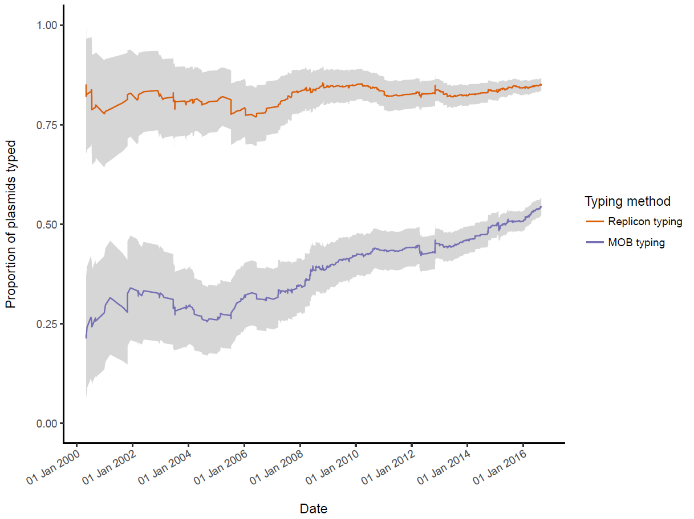

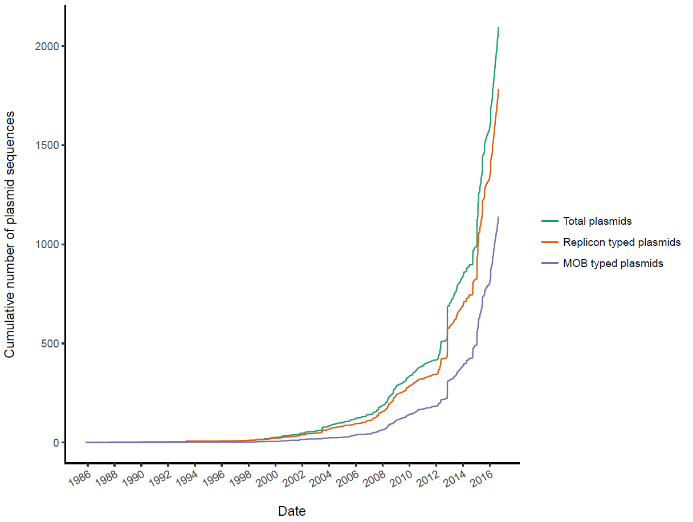


**A**

**B**

Figure S8: Plasmid typing by date; MOB typing using alternative set of MOB queries. (A) shows cumulative numbers of plasmids typed; (B) shows proportion of plasmids typed. For details see corresponding legend, Figure 2.

### Supplementary Figure S9


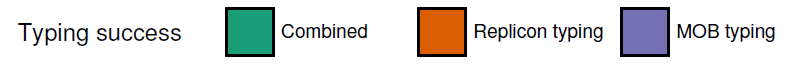

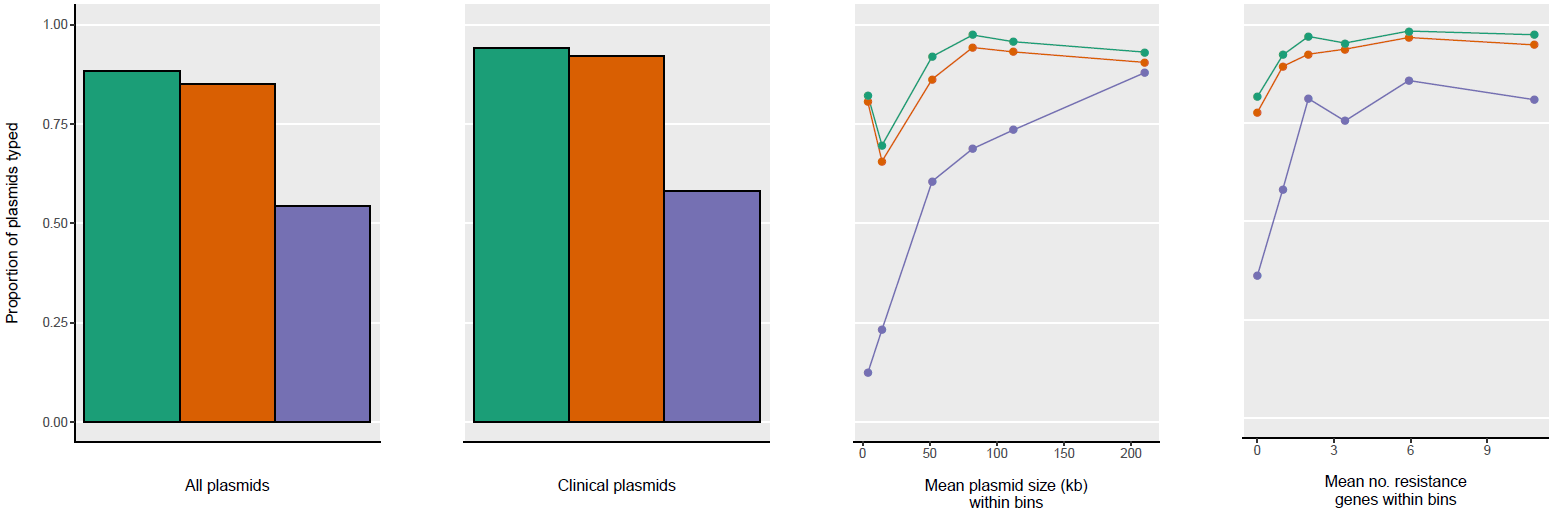


Figure S9: Relationship between typing success and plasmid characteristics; MOB typing using alternative set of MOB queries. For details, see corresponding legend, Figure 3.

### Supplementary Figure S10

**A**

**B**


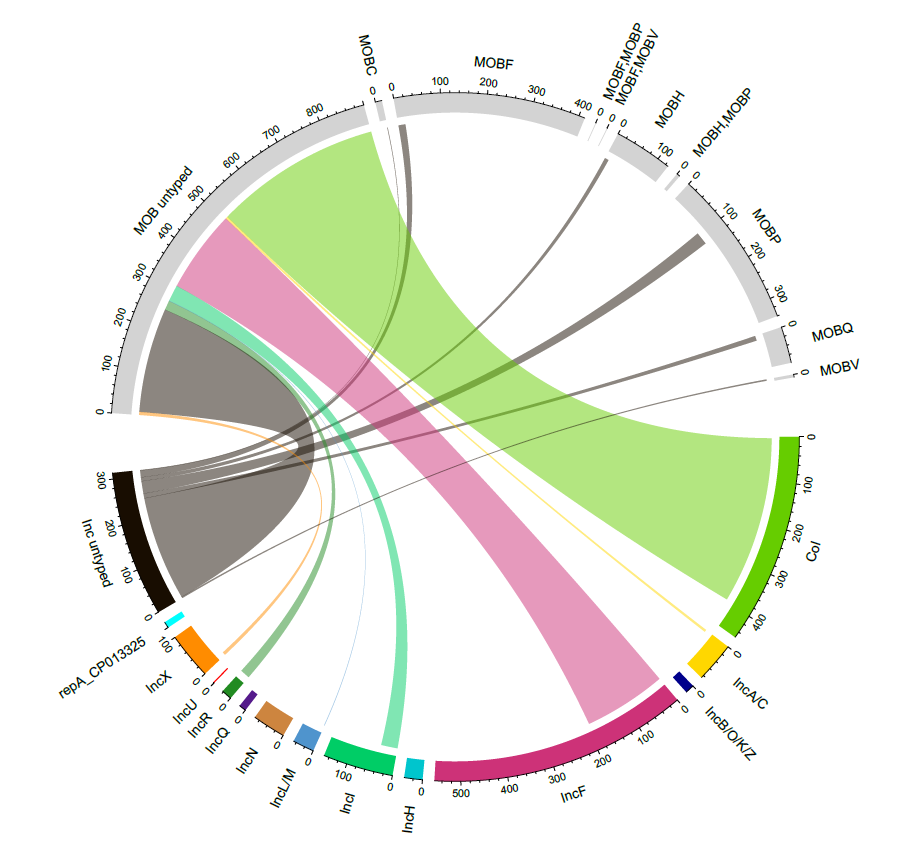

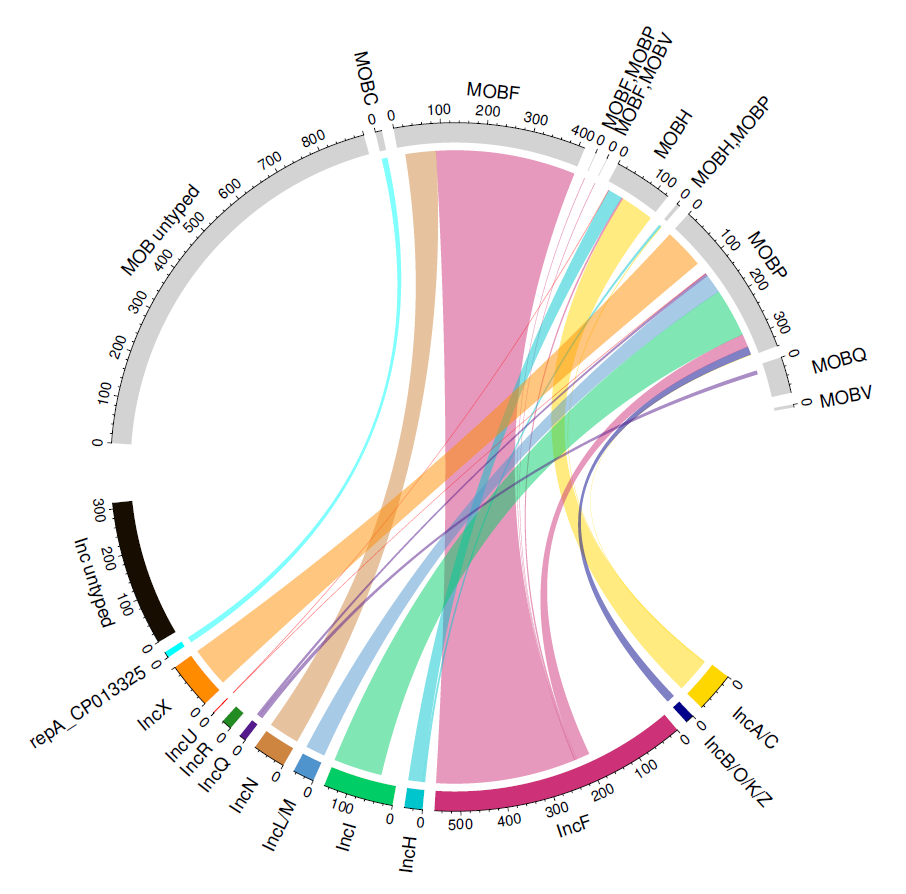


Figure S10: Chord diagrams illustrate associations between replicon families and MOB types; MOB typing using alternative set of MOB queries. (A) Replicon family-MOB type associations amongst plasmids un-typed by one or both schemes. (B) Replicon family-MOB type associations amongst plasmids with both replicon and MOB type detected. Data on Col plasmids are deliberately not shown (see main text). For details, see corresponding legend, Figure 4.

### Supplementary Figure S11


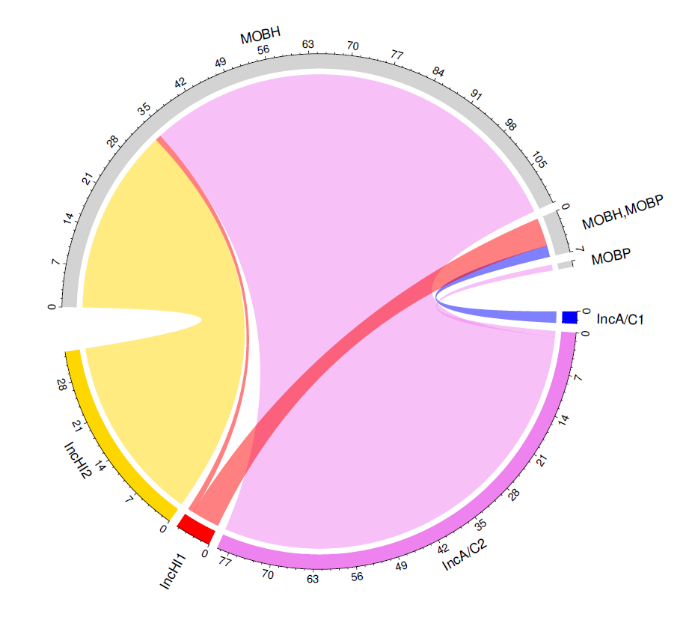

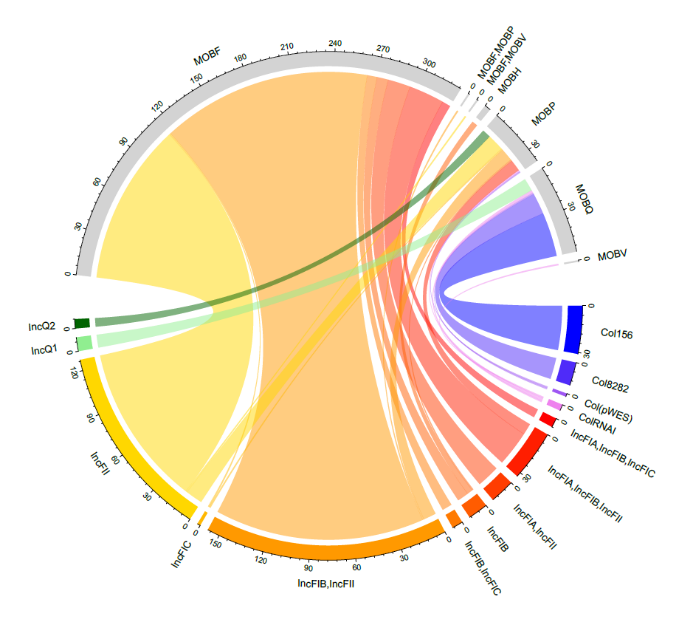


**A**

**B**


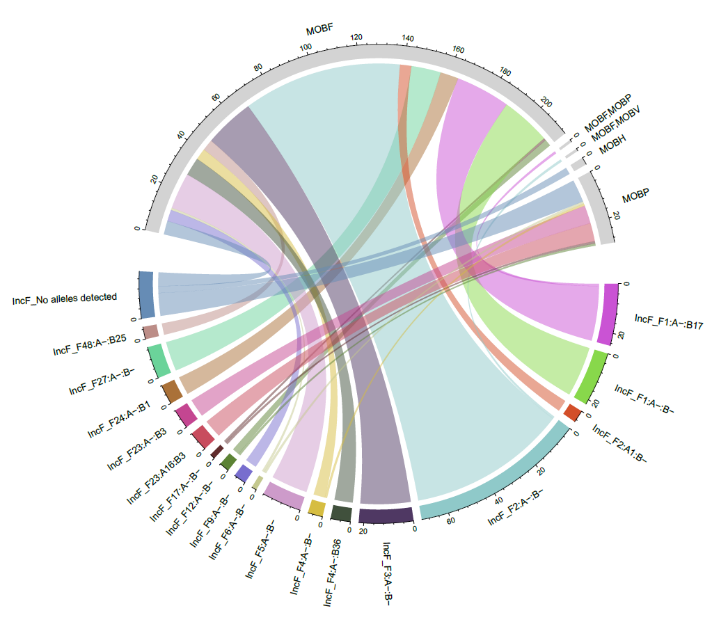

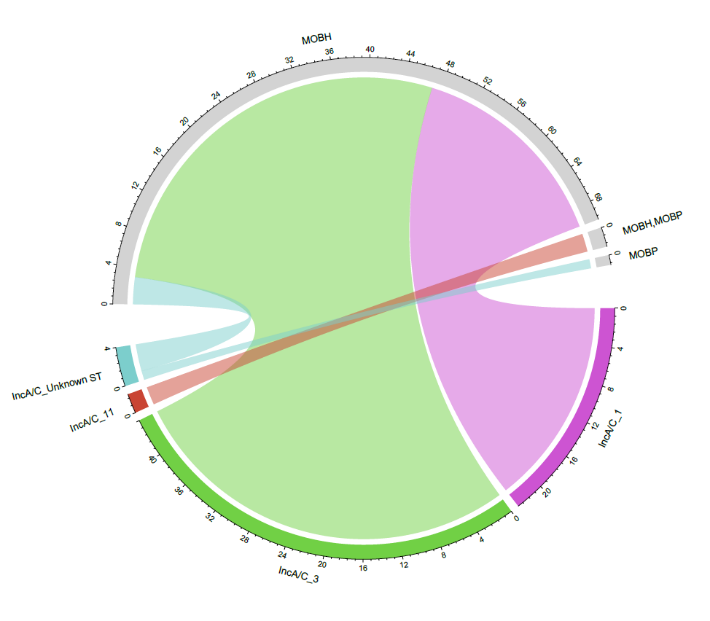


**C**

**D**

Figure S11: Chord plots A and B illustrate associations between replicon and MOB types, for replicon types belonging to non-concordant replicon families. Where replicon types are shown to be non-concordant and a pMLST scheme is available, chord plots C and D show associations between pMLST types and MOB types. MOB typing was conducted using the alternative set of MOB queries. For details see corresponding legend, Figure 5.
