# Supplementary material for: Ordering the mob: Insights into replicon and MOB typing schemes from analysis of a curated dataset of publicly available plasmids
Source: Plasmid. 2017 May;91:42–52. doi: 10.1016/j.plasmid.2017.03.002 (PMC5466382; doi:10.1016/j.plasmid.2017.03.002)
Supplement: Supplementary methods S1 — Methods for compiling the curated plasmid dataset are described. In addition, details on downloaded datasets used for plasmid typing and MLST are provided. MOB typing methods are outlined, including the sequences of query proteins, and bioinformatic procedures for selecting best PSI-BLAST hits. [file mmc2.docx]

## Supplementary Methods S1

**Entrez query used for initial retrieval of putative complete plasmid accessions from NCBI:**

Entrez.esearch(db="nucleotide", term=str("Enterobacteriaceae[porgn] AND biomol_genomic[PROP] AND plasmid[filter] NOT complete cds[Title] NOT gene[Title] NOT genes[Title] NOT contig[Title] NOT scaffold[Title] NOT whole genome map[Title] NOT partial sequence[Title] NOT partial plasmid[Title] NOT locus[Title] NOT region[Title] NOT fragment[Title] NOT integron[Title] NOT transposon[Title] NOT insertion sequence[Title] NOT insertion element[Title] NOT phage[Title] NOT operon[Title]")

**Filtering non-plasmid or non-complete plasmid accessions**

The following regular expression term was used to filter non-plasmid and non-compete plasmid accessions based on the description in the accession title:

'contig|\sgene(?!tic|ral|rat|ric)|integron|transposon|scaffold|insertion sequence|insertion element|phage|operon|partial sequence|partial plasmid|region|fragment|locus|complete (?!sequence|genome|plasmid|\.|,)|(?<!complete sequence, )whole genome shotgun|artificial|synthetic|vector'

Having excluded accessions using the above term, the following term was used as an inclusion criterion for the remaining accessions:

'complete(?= sequence| genome| plasmid)'

For accessions matching neither exclusion nor inclusion criteria, MLST typing was conducted to exclude chromosomal accessions, and manual filtering was conducted, focusing on the tails of the size distribution.

**Downloaded datasets**

MLST alleles were downloaded from <http://pubmlst.org/data/> on 22^nd^ February 2017. For *Escherichia coli*, MLST scheme 1 was used.

The PlasmidFinder database was downloaded from <http://www.genomicepidemiology.org/> on 20^th^ April 2016.

pMLST alleles and associated profiles (used to determine sequence type) were downloaded from <http://pubmlst.org/plasmid/> on 25^th^ November 2016. For IncF plasmids, pMLST sequence type is determined according to the FAB formula rather than a downloaded profile. The FAB formula means sequence types are determined according to the allele for IncFII, IncFIA and IncFIB. As an example, the IncF sequence type F2:A-:B- would reflect detection of allele 2 for IncFII, and no allele detection for IncFIA and IncFIB.

The ResFinder database was downloaded from <http://www.genomicepidemiology.org/> on 3^rd^ September 2016.

**MOB protein queries used in PSI-BLAST searches**

The following six MOB proteins were used as queries in PSI-BLAST searches used to assign MOB types to plasmids: MobC_CloDF13 (MOBC), TrwC_R388 (MOBF), TraI_R27 (MOBH), TraI_RP4 (MOBP), MobA_RSF1010 (MOBQ), MobM_pMV158 (MOBV). Their N-terminal sequences are shown below. The primary MOB typing results presented in the main text are based on queries with these proteins, which have also been used previously as queries by Garcillán-Barcia et al. (2009) and Smillie et al. (2010).

>MOBC|CAB62410.1_mobC__plasmid__[Enterobacter_cloacae]

MALERYNVSHAKRQARNAEKTRLTLRWLREELCSTAELVARRLGIAAVQPVYRFLDSLVA

KGLLVRAKYPVDGRQVSVWGLTPHGVAFSFDEDEPLTDIIPFQPSRVSAAQLPHRLAVQS

LRLAMEARGATGWRYLHRIALKGMKVPDALAELDGRTVAFEVERTVKSRRRYQEVVSGYL

FNRRANGIDEIWYICPDRATQVRVQRAILSVDEIVNPQTGEARKTAELDRERLFACFKFM

TTE

>MOBF|FAA00039.1_TPA:_TrwC_protein__plasmid__[Escherichia_coli]

MLSHMVLTRQDIGRAASYYEDGADDYYAKDGDASEWQGKGAEELGLSGEVDSKRFRELLA

GNIGEGHRIMRSATRQDSKERIGLDLTFSAPKSVSLQALVAGDAEIIKAHDRAVARTLEQ

AEARAQARQKIQGKTRIETTGNLVIGKFRHETSRERDPQLHTHAVILNMTKRSDGQWRAL

KNDEIVKRTRYLGAVYNAELAHELQKLGYQLRYGKDGNFDLAHIDRQQIEGFSKRTEQIA

EWYAARGLDPNSVSLEQKQAAKVLSRAKKTSVDREALRAEWQATAKELGIDFSRREWSGR

>MOBH|NP_058333.1_hypothetical_protein_R27_p120__plasmid__[Salmonella_enterica_subsp._enterica_serovar_Typhi]

MNFRALFLSMQRVFGIFSRRENDVSELMMKDAANFSPFAQIIGEQKYTVPDHPNPEVLKF

IEYPTRPAGIQTFNEQSILSLYRDKLHSISMMLAISDGDIREDAYTFTNLVLKPLIEYIR

WIHLLPASENHHHNGIGGLLSHSLEVAMISLKNANHSELRPIGYQDEEVVRRKVYLYAAF

ICGLVHDAGKVYDLDIVSLNLSETLTWAPSSQSLLDWARENNVVEYEIHWRKRIHNQHNI

WSSVFLERILDPVCMSFLDRVKKERVYAKMVTALNVYNDGNDFLSKCVRTSDYYSTGTDL

>MOBP|WP_011205818.1_MULTISPECIES:_protein_TraI_[Proteobacteria]

MIAKHVPMRSIKKSDFAELVKYITDEQGKTERLGHVRVTNCEANTLPAVMAEVMATQHGN

TRSEADKTYHLLVSFRAGEKPDAETLRAIEDRICAGLGFAEHQRVSAVHHDTDNLHIHIA

INKIHPTRNTIHEPYRAYRALADLCATLERDYGLERDNHETRQRVSENRANDMERHAGVE

SLVGWIKRECLPELQAAQSWEDLHRVLRENGLKLRERGNGFIFEAGDGTTVKASTVSRDL

SKPKLEARFGAFTPAEGGEAPRRREYRAKPLKTRIDTTELYARYQSERQEMGAVRKGELD

>MOBQ|NP_044304.1_mobilization_protein_A__plasmid__[Escherichia_coli]

MAIYHLTAKTGSRSGGQSARAKADYIQREGKYARDMDEVLHAESGHMPEFVERPADYWDA

ADLYERANGRLFKEVEFALPVELTLDQQKALASEFAQHLTGAERLPYTLAIHAGGGENPH

CHLMISERINDGIERPAAQWFKRYNGKTPEKGGAQKTEALKPKAWLEQTREAWADHANRA

LERAGHDARIDHRTLEAQGIERLPGVHLGPNVVEMEGRGIRTDRADVALNIDTANAQIID

LQEYREAIDHERNRQSEEIQRHQRVSGADRTAGPEHGDTGRRSPAGHEPDPAGQRGAGGG

>MOBV|AAA25387.1_mobilization_peptide__plasmid__[Plasmid_pMV158]

MSYMVARMQKMKAGNLGGAFKHNERVFETHSNKDINPSRSHLNYELTDRDRSVSYEKQIK

DYVNENKVSNRAIRKDAVLCDEWIITSDKDFFEKLDEEQTRTFFETAKNYFAENYGESNI

AYASVHLDESTPHMHMGVVPFENGKLSSKAMFDREELKHIQEDLPRYMSDHGFELERGKL

NSEAKHKTVAEFKRAMADMELKEELLEKYHAPLFVDERTGELNNDTEAFWHEKEFADMFE

VQSPIRETTNQEKMDWLRKQYQEELKKLESSKKPLEDDLSHLEELLDKKTKEYIKIDSEA

To examine the robustness of MOB typing results, we ran PSI-BLAST searches using the same methodology, except with different MOB protein queries representing each family. Initially, these were: TraX_pAD1 (MOBC), TraI_F (MOBF), TraI_pIP1202 (MOBH), TraS_pSB102 (MOBP), TraA_pTi (MOBQ), Mob_pBBR1 (MOBV). Garcillán-Barcia et al. (2009) suggest these proteins can be used as alternative relaxase prototypes for MOB typing. However, the MOBC TraX_pAD1 query, known to be present in plasmids from Firmicutes taxa, retrieved no hits at a relatively relaxed E-value threshold of 0.01. We instead selected accession WP_020316827.1 as a MOBC query protein. This accession was chosen from a list of proteins that had been MOB typed using Profile hidden Markov model searches (Guglielmini et al., 2011); the list was kindly provided by Professor Eduardo P. C. Rocha. Although annotated as a hypothetical protein in NCBI, it shows 27% sequence identity to the original MOBC prototype (CAB62410.1), and is present on plasmids, suggesting that it is a genuine MOBC relaxase. The N-terminal sequences of the alternative set of MOB queries are shown below.

>MOBC|WP_020316827.1_MULTISPECIES:_hypothetical_protein_[Enterobacteriaceae]

MNTFDINAQKREKIVIWLAKFGFSTRDLLSKMLGVNVDGQGAFFKKLVESGITKEEYVPG

TRKRVITLTPDGVQQARIYQPDLEVKALRKFPLHTLIHSYSIQSFLTTQKGVKDFFSETE

LAKRKFIRRPDLLIVNDAGVKIAIEVELTQKDVNRVYFNFYGHVQDWQQERIDHVIYLFS

SPTVLARYEELYRKNPWPKFITTDGNVRHMSRSGSVDPSHAHTHGLMYFHKFEPYAL

>MOBF|P14565.2_RecName:_Full=Multifunctional_conjugation_protein_TraI;_Includes:_RecName:_Full=DNA_relaxase_TraI;_AltName:_Full=DNA_nickase_TraI;_AltName:_Full=Transesterase_TraI;_Includes:_RecName:_Full=DNA_helicase_I

MMSIAQVRSAGSAGNYYTDKDNYYVLGSMGERWAGRGAEQLGLQGSVDKDVFTRLLEGRL

PDGADLSRMQDGSNRHRPGYDLTFSAPKSVSMMAMLGGDKRLIDAHNQAVDFAVRQVEAL

ASTRVMTDGQSETVLTGNLVMALFNHDTSRDQEPQLHTHAVVANVTQHNGEWKTLSSDKV

GKTGFIENVYANQIAFGRLYREKLKEQVEALGYETEVVGKHGMWEMPGVPVEAFSGRSQT

IREAVGEDASLKSRDVAALDTRKSKQHVDPEIKMAEWMQTLKETGFDIRAYRDAADQRAD

>MOBH|YP_001101767.1_type_IV_conjugative_transfer_system_protein_TraI__plasmid__[Yersinia_ruckeri]

MLKALNKLFGGRSGVIETAPSARVLPLKDVEDEEIPRYPPFAKGLPVAPLDKILATQAEL

IEKVRNSLGFTVDDFNRLVLPVIQRYAAFVHLLPASESHHHRGAGGLFRHGLEVAFWAAQ

ASESVIFSIEGTPRERRDNEPRWRLASCFSGLLHDVGKPLSDVSITDKDGSITWNPYSES

LHDWAHRHEIDRYFIRWRDKRHKRHEQFSLLAVDRIIPAETREFLSKSGPSIMEAMLEAI

SGTSVNQPVTKLMLRADQESVSRDLRQSRLDVDEFSYGVPVERYVFDAIRRLVKTGKWKV

>MOBP|CAC79161.1_TraS_protein__plasmid__[uncultured_bacterium]

MAKREIDGVLKDWGERVDYGRVQGRKGKNIAGGRYSKPAPEKKPSGREKLEATVRKAPEV

MVKISGGGKDMRSIKAHMDYISRNGAVELEDEQGRVHQGKEDVRAVRDDWRGGGIPYEDG

TKREAFNIVLSMPPGTNRQAVKDAARAFAAELFGNHQYVFAAHDDEKHPHVHLAVKAVDL

DGVRLNPRKADLQNWRETFAEKLRAQGIDANATPRKARGIVRKAEKQAVVHIEQRGKTPR

VKASRRADAEREASGVAVKPNPAQDRISVTRKDVQRAYGTAARALAKGEAKDKQLAVEIV

>MOBQ|AAC17212.1_TraA__plasmid__[Agrobacterium_fabrum_str._C58]

MAIAHFSASIVSRGDGRSVVLSAAYQHCAKMEYEREARTIDYTRKQGLVHQEFILPADAP

KWVRALIADCSVAGASEAFWNKVEAFEKRSDAQLARDLTIALPRELTSEQNIALVRDFVE

KHILGKGMVADWVYHDNPGNPHIHLMTTLRPLTEDGFGAKKVAVIGEDGQLVRTKSGKIL

YELWAGSTDDFNVVRDGWFERLNHHLTLGGIDLKIDGRSYEKQGIDLEPTIHLGVGAKAI

SRKAEQQGVRPELERIELNEERRSENTRRILKNPAIVLDLIMREKSVFDERDVAKVLHRY

>MOBV|Q9Z5R6_Mob_protein

MAAYAIMRCKKLAKMGNVAASLKHAYRERETPNADASRTPENEHWAASSTDEAMGRLREL

LPEKRRKDAVLAVEYVMTASPEWWKSASQEQQAAFFEKAHKWLADKYGADRIVTASIHRD

ETSPHMTAFVVPLTQDGRLSAKEFIGNKAQMTRDQTTFAAAVADLGLQRGIEGSKARHTR

IQAFYEALERPPVGHVTISPQAVEPRAYAPQGLAEKLGISKRVETPEAVADRLTKAVRQG

YEPALQAAAGAREMRKKADQAQETARDLRERLKPVLDALGPLNRDMQAKAAAIIKAVGEK

**Optimising the PSI-BLAST E-value threshold**

We initially conducted MOB typing with E-value thresholds used previously (1e-4 except for MOBF and MOBV alignments where more stringent thresholds of 1e-8 and 1e-5 were applied) (Garcillán-Barcia et al., 2009). We compared our MOB typing results with those of previous authors (see Supplementary Table S1 in Smillie et al., 2010). This demonstrated discrepancies in typing, with a tendency towards false negatives (i.e. not assigning a MOB type when a MOB type was assigned by Smillie *et al.*). As mentioned in the main text, E-values are inflated when a larger database is searched. We hypothesised that compared with our dataset, previous authors searched smaller databases (leading them to obtain more significant E-values), and this could at least partly explain the discrepancies. We therefore adjusted the E-value threshold for MOB query alignments, from 1e-4 through to 10, in increments of 1 decimal place. We found that the following E-value thresholds minimised discrepancies with typing results of Smillie *et al.*: MOBC, 0.001; MOBF, 0.01; MOBH, 0.01; MOBP, 1; MOBQ, 0.0001; MOBV, 0.01. These thresholds were also found to be optimal when using the alternative set of MOB queries. During optimisation, as well as comparing our typing results to those of Smillie *et al.*, discrepancies in hits retrieved at different E-value thresholds were also examined; the plausibility of discrepant hits was assessed to guide optimisation. Specifically, we examined the NCBI coding sequence annotation associated with the coding region at which the hit intersected (see ‘PSI-BLAST best hit selection’ below). For further details, see Methods S2. The same approach was used to optimise the E-value threshold when conducting MOB typing on plasmids analysed by Shintani et al. (2015). We found that the E-values used by Garcillán-Barcia et al. (2009) were optimal in the case of the Shintani *et al.* dataset.

In addition to the influence of database size on calculated E-values, other potential explanations for MOB typing discrepancies compared with findings of previous authors are as follows: 1. potential differences in methods used for selecting best hits (to our knowledge, previous authors do not provide methods). 2. PSI-BLAST search profiles depend on database content, since information about amino acid conservation at different positions is derived from query–database alignments and used to guide profile searches on the subsequent iteration (Chen et al., 2016). In a scenario where two databases of the same size but containing different sequence content were searched, a sequence shared between the two databases may not both be assigned a given MOB type due to the different search profiles generated.

**PSI-BLAST best hit selection**

Stringent filtering was used to select best hits as follows: alignment positions on the translated plasmid sequences were mapped back to nucleotide positions; translations produced from the reverse complement DNA strand were mapped back to the original strand; and at nucleotide loci encoding multiple proteins that were BLAST hits, a single best hit was selected (that is, a given nucleotide locus was assumed to encode a single relaxase protein, even if hits were produced from both the original and reverse complement strands). Best hits were selected amongst hits intersecting the same coding region (according to NCBI CDS feature annotation). In cases where there was no intersection with an annotated coding region, the best hit was selected amongst overlapping hits (defined stringently as alignments showing any degree of overlap). Best hits were selected according to the iteration at which they were produced (favouring lower iterations, with the aim of minimising the impact of false-positive hits which can corrupt subsequent iterations), and secondly according to percentage identity and coverage. To further understand the selection of best PSI-BLAST hits, see Supplementary Table S2 (sheet 3) which shows raw PSI-BLAST output, as well as assigned MOB types, following best hit selection.

**References**

Chen, J., Guo, M., Wang, X., and Liu, B. (2016). A comprehensive review and comparison of different computational methods for protein remote homology detection. *Brief. Bioinform.*, 1–14. doi:doi: 10.1093/bib/bbw108.

Garcillán-Barcia, M. P., Francia, M. V., and De La Cruz, F. (2009). The diversity of conjugative relaxases and its application in plasmid classification. *FEMS Microbiol. Rev.* 33, 657–687. doi:10.1111/j.1574-6976.2009.00168.x.

Guglielmini, J., Quintais, L., Garcillán-Barcia, M. P., de la Cruz, F., and Rocha, E. P. C. (2011). The repertoire of ice in prokaryotes underscores the unity, diversity, and ubiquity of conjugation. *PLoS Genet.* 7. doi:10.1371/journal.pgen.1002222.

Shintani, M., Sanchez, Z. K., and Kimbara, K. (2015). Genomics of microbial plasmids: Classification and identification based on replication and transfer systems and host taxonomy. *Front. Microbiol.* 6, 1–16. doi:10.3389/fmicb.2015.00242.

Smillie, C., Garcillan-Barcia, M. P., Francia, M. V, Rocha, E. P., and de la Cruz, F. (2010). Mobility of plasmids. *Microbiol Mol Biol Rev* 74, 434–452. doi:10.1128/MMBR.00020-10.
