# Supplementary material for: Ordering the mob: Insights into replicon and MOB typing schemes from analysis of a curated dataset of publicly available plasmids
Source: Plasmid. 2017 May;91:42–52. doi: 10.1016/j.plasmid.2017.03.002 (PMC5466382; doi:10.1016/j.plasmid.2017.03.002)
Supplement: Table S4 — The zip file contains a spreadsheet file showing the conserved domains associated with replication proteins of the PlasmidFinder database. The relationship between replicon types in terms of sharing of replication protein domains is visualised using networks. The networks are provided as separate files within the zip file. The interactive network (html file) opens in a web browser and is a bipartite network - one set of nodes represents replicon types, another set of nodes represents conserved domains. The other network is a static network (PDF file) derived from the bipartite network; it shows replicon type nodes only. [file mmc7.zip › static_network.pdf]

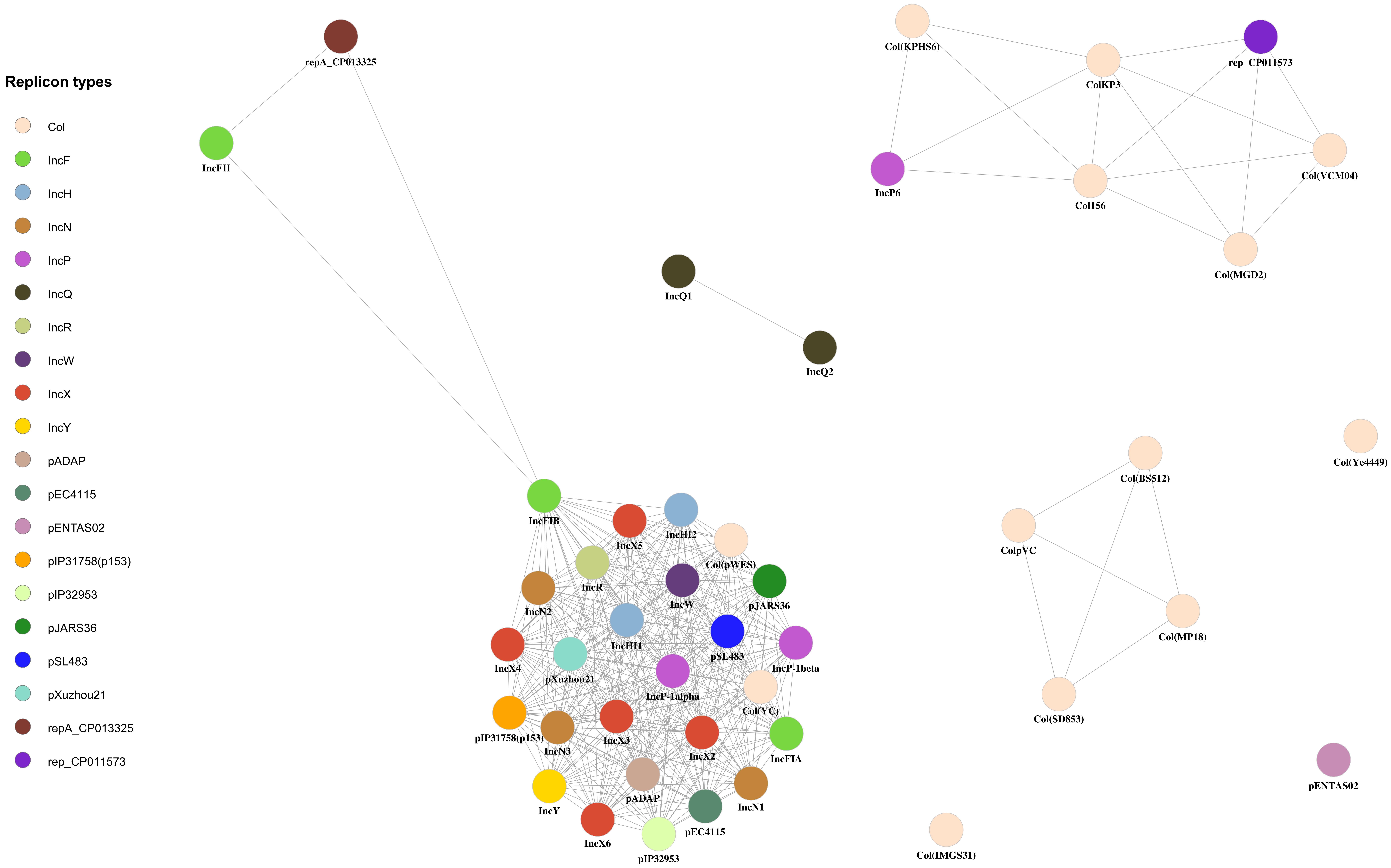

Replicon types
Col
IncF
IncH
IncN
IncP
IncQ
IncR
IncW
IncX
IncY
pADAP
pEC4115
pENTAS02
pIP31758(p153)
pIP32953
pJARS36
pSL483
pXuzhou21
repA_CP013325
rep_CP011573
repA_CP013325
IncFII
IncQ1
IncQ2
Col(KPHS6)
ColKP3
rep_CP011573
IncP6
Col156
Col(VCM04)
Col(MGD2)
IncFIB
IncX5
IncHI2
Col(pWES)
IncR
IncN2
IncW
pJARS36
IncHI1
pSL483
IncP-1beta
IncX4
pXuzhou21
IncP-1alpha
Col(YC)
pIP31758(p153)
IncN3
IncX3
IncX2
IncFIA
IncY
pADAP
IncN1
IncX6
pEC4115
pIP32953
Col(BS512)
Col(Ye4449)
ColpVC
Col(MP18)
Col(SD853)
pENTAS02
Col(IMGS31)
